# The dynamical organization of the core pluripotency transcription factors responds to differentiation cues in early S-phase

**DOI:** 10.3389/fcell.2023.1125015

**Published:** 2023-05-04

**Authors:** Camila Oses, Marcos Gabriel Francia, Paula Verneri, Camila Vazquez Echegaray, Alejandra Sonia Guberman, Valeria Levi

**Affiliations:** ^1^ Instituto de Química Biológica de la Facultad de Ciencias Exactas y Naturales (IQUIBICEN), Facultad de Ciencias Exactas y Naturales, CONICET-Universidad de Buenos Aires, Buenos Aires, Argentina; ^2^ Departamento de Fisiología, Biología Molecular y Celular, Facultad de Ciencias Exactas y Naturales, Universidad de Buenos Aires, Buenos Aires, Argentina; ^3^ Departamento de Química Biológica, Facultad de Ciencias Exactas y Naturales, Universidad de Buenos Aires, Buenos Aires, Argentina

**Keywords:** pluripotency transcription factors, DNA replication, embryonic stem cells, cell cycle, differentiation, condensates

## Abstract

DNA replication in stem cells is a major challenge for pluripotency preservation and cell fate decisions. This process involves massive changes in the chromatin architecture and the reorganization of many transcription-related molecules in different spatial and temporal scales. Pluripotency is controlled by the master transcription factors (TFs) OCT4, SOX2 and NANOG that partition into condensates in the nucleus of embryonic stem cells. These condensates are proposed to play relevant roles in the regulation of gene expression and the maintenance of pluripotency. Here, we asked whether the dynamical distribution of the pluripotency TFs changes during the cell cycle, particularly during DNA replication. Since the S phase is considered to be a window of opportunity for cell fate decisions, we explored if differentiation cues in G1 phase trigger changes in the distribution of these TFs during the subsequent S phase. Our results show a spatial redistribution of TFs condensates during DNA replication which was not directly related to chromatin compaction. Additionally, fluorescence fluctuation spectroscopy revealed TF-specific, subtle changes in the landscape of TF-chromatin interactions, consistent with their particularities as key players of the pluripotency network. Moreover, we found that differentiation stimuli in the preceding G1 phase triggered a relatively fast and massive reorganization of pluripotency TFs in early-S phase. Particularly, OCT4 and SOX2 condensates dissolved whereas the lifetimes of TF-chromatin interactions increased suggesting that the reorganization of condensates is accompanied with a change in the landscape of TF-chromatin interactions. Notably, NANOG showed impaired interactions with chromatin in stimulated early-S cells in line with its role as naïve pluripotency TF. Together, these findings provide new insights into the regulation of the core pluripotency TFs during DNA replication of embryonic stem cells and highlight their different roles at early differentiation stages.

## Introduction

Pluripotent stem cells (PSCs) constitute a promise for both regenerative medicine and disease modeling (Doss and Sachinidis, 2019; Grandy et al., 2019; Suman et al., 2019; Yamanaka, 2020) due to their capabilities to self-renew indefinitely and differentiate into all cell types derived from the three germ layers.

Pluripotency is controlled by a regulatory network directed by the master transcription factors (TFs) OCT4 (also known as POU5F1), SOX2 and NANOG which ultimately induces genes that promote self-renewal and represses those involved in differentiation (Loh et al., 2006). NANOG is a naïve pluripotency TF (Martello and Smith, 2014) since it promotes the undifferentiated state of the cells even in the absence of pluripotency signals (Chambers et al., 2003) and it is downregulated at early stages of differentiation (Kalkan et al., 2017). On the other hand, OCT4 and SOX2 are general pluripotency TFs that are also expressed during primed pluripotency (Kalkan et al., 2017). Additionally, their levels should be constrained for efficient self-renewal since unbalances lead to differentiation (Niwa et al., 2000; Masui et al., 2007).

In the last years, the cell cycle has come under the spotlight for its role in pluripotency maintenance and cell fate decisions (Coronado et al., 2013; Neganova et al., 2014; Gonzales et al., 2015; Boward et al., 2016; Soufi and Dalton, 2016; Liu et al., 2019). Strikingly, the cell cycle of PSCs is faster than that of somatic cells (11–16 h vs. 24–32 h, respectively) (Becker et al., 2006; Calder et al., 2013; Cannon et al., 2015; Waisman et al., 2019) and presents a very short G1 phase (Waisman et al., 2017; Zaveri and Dhawan, 2018).

We have previously shown that the inhibition of DNA replication interferes with the transcriptional switch required for the transition from naïve to primed pluripotency (Waisman et al., 2017). Further works identified G1 as the critical “window of opportunity” when PSCs are sensitive to signals that induce changes in the gene expression program required to switch from pluripotency to differentiation (Sela et al., 2012; Coronado et al., 2013; Pauklin and Vallier, 2013). Particularly, cells in G1 respond immediately to differentiation cues whereas those that are transiting through S or G2 phases do not respond until the next cell cycle, most certainly in the following G1 phase (Sela et al., 2012; Coronado et al., 2013; Pauklin and Vallier, 2013).

High throughput technologies such as chromosome conformation capture methods shed some light on the changes in the chromatin landscape during the cell cycle (Dalton, 2015; Ma et al., 2015; Soufi and Dalton, 2016). Particularly, it was demonstrated that chromatin structures with different hierarchies -such as compartments, small/big topologically associating domains (TADs) and loops-reassemble in different time windows upon mitosis exit. For example, genome active and inactive compartments start their assembly in G1 and increase in strength through S and G2 before dissolution in mitosis whereas TADs insulation has its maximum at G1 phase and declines during replication until its minimum at G2 (Nagano et al., 2017). Additionally, long-range chromatin contacts are reestablished after mitosis in complex and asynchronous patterns that do not correlate directly with the transcriptional reactivation of the involved elements (Pelham-Webb et al., 2021).

Moreover, DNA replication requires changes in DNA accessibility that affect the chromatin organization during S phase in different time and spatial scales. For example, the passage of the replication fork temporarily affects nucleosome assembly perturbing chromatin organization in the nanometer-scale (Stewart-Morgan et al., 2020). Also, the condensation and insulation of specific TADs progressively change during this phase with a dynamic that depends on their replication time (Nagano et al., 2017).

In contrast, few works have addressed how pluripotency TFs respond to the profound and asynchronous changes occurring during the cell cycle. Particularly, NANOG levels significantly drop down whereas SOX2 and OCT4 levels persist during mitosis and seem to play relevant roles for chromatin organization (Liu et al., 2017). In mitosis, SOX2 and OCT4 bind to chromosomes and the mitotic bookmarking role of SOX2 is relevant for pluripotency and differentiation (Deluz et al., 2016). On the other hand, SOX2/OCT4 ratio in G1 phase impacts on cell fate commitment (Strebinger et al., 2019). Additionally, OCT4 is essential for the re-establishment of chromatin organization after mitosis and for promoting chromatin accessibility during interphase (Friman et al., 2019). In this line, OCT4 and SOX2 were identified as pioneer TFs and this activity seem to be fundamental for reprogramming and differentiation (Iwafuchi-Doi and Zaret, 2014; Jerabek et al., 2014; Soufi et al., 2015; King and Klose, 2017; Roberts et al., 2021). Further evidence showed that the pioneer activity of these TFs is also relevant for pluripotency preservation (Friman et al., 2019; Maresca et al., 2022).

Altogether, these works highlight the role of OCT4 and SOX2 in modulating the chromatin accessibility landscape and emphasize the necessity of considering the cell phase as a relevant regulatory layer when studying pluripotency maintenance and cell fate commitment.

We have previously observed that OCT4 and SOX2 distribute between the nucleoplasm and condensates or foci in mouse embryonic stem cells (ESCs) (Verneri et al., 2020). Relevantly, the nuclear distributions of these pluripotency TFs change during early differentiation stages preceding their downregulation and this spatial reorganization is accompanied by modifications in TF-chromatin interactions (Verneri et al., 2020). Further evidence suggests that OCT4 forms liquid condensates in these cells which are involved in the remodeling of TADs, contributing to cell fate decisions (Wang et al., 2021).

Here, we asked whether the nuclear organization of the core pluripotency TFs changes during the cell cycle and specifically, during S phase. We mentioned before that the G1 phase is a window of opportunity in which the cell is able to receive differentiation instructions and that blockage of DNA replication impairs the transcriptional switch required to initiate the differentiation process. Then, we reasoned that S phase could be a window of opportunity to execute changes in the chromatin landscape and the dynamical distribution of the TFs required for cell fate transitions.

We show that OCT4 and SOX2 foci remodel during the early-S to mid-S transition in which these active genes are expected to be replicated. Relevantly, this reorganization does not occur concomitantly to changes in the chromatin condensation assessed by HP1α distribution. Moreover, fluorescence correlation spectroscopy (FCS) revealed that the landscape of interactions of these TFs respond to the cell cycle in a TF-specific manner. Finally, we report that the organization and dynamics of these pluripotency TFs in early-S phase respond to differentiation signals received in the preceding G1 phase.

## Materials and methods

### Cell culture and differentiation

The experiments were performed using the mouse ESC line W4 (provided by the Rockefeller University Core Facility), the YPet-OCT4 and YPet-SOX2 ESC lines previously generated in our laboratory from the W4 cell line (Verneri et al., 2020) and the eGFP-NANOG cell line that was generated in this work. ESCs were maintained on 0.1% gelatin-coated dishes, passed every 3 days using trypsin-EDTA (Gibco) and grown at 37°C in a 5% CO_2_ (v/v) incubator. Cells were cultured in DMEM (Gibco) supplemented with 15% ESC qualified fetal bovine serum (Gibco), 100 mM MEM nonessential amino acids (Gibco), 2 mM l-alanyl-L-glutamine (Gibco), 0.5 mM 2-mercaptoethanol, 100 U/mL penicillin (Gibco), 100 mg/mL streptomycin (Gibco), leukemia inhibitory factor (LIF) and 2i [1 μM PD0325901 (Tocris) and 3 μM CHIR99021 (Tocris)].

To induce non-directed differentiation, ESCs were incubated in the absence of LIF and 2i during 4 h for induced early-S phase cells measurements and for 24 or 48 h for eGFP-NANOG stable cell line validation.

### Cells preparation for imaging experiments

For microscopy measurements, 18-mm round coverslips were placed into the wells of a 12 multiwell plate, incubated for 1 h with a 100 μg/mL poly-D-Lysine (Sigma) and for 2 h with a solution 20 μg/mL laminin (Thermo Fisher) at 37°C in a 5% CO_2_ incubator. Next, ∼75,000 cells were added in each well and incubated with culture medium.

Transient transfection of cells with PCNA-RFP and/or HP1α-eGFP vectors was performed using Lipofectamine 2000 (Thermo Fisher) and 1.6 µg of plasmid DNA in Opti-MEM medium (Thermo Fisher) after culturing the cells for 24 h. The transfection medium was replaced after 6 h with fresh culture medium. PCNA-RFP coding vector (Sporbert et al., 2005) was kindly provided by Dr. Cristina Cardoso. Microscopy experiments were run after 48 h from transfection.

For the induction of YPet-OCT4 and YPet-SOX2 expression, the corresponding ESCs were incubated with 5 μg/mL doxycycline (Dox) for 48 h prior to microscopy measurements (Verneri et al., 2020).

### Generation of the eGFP-NANOG ESC line

eGFP-NANOG coding sequence, kindly provided by Dr. Nicolas Platcha, was subcloned in a PiggyBac transposon vector generating the PB-eGFP-NANOG plasmid. Cells were plated during 24 h onto a 0.1% gelatin-coated dish and co-transfected, using Lipofectamine 2000 (Thermo Fisher) as described above, with PB-eGFP-NANOG and the corresponding transposase coding vector in a 3:1 relation. After 72 h of expression, puromycin (1 μg/μL) was added. eGFP-NANOG expression was verified by fluorescence microscopy after 72 h from selection.

Clone isolation was performed manually, picking the colonies and re-plating them separately in a 24-well plate. After amplification of each clone, validation was performed by analysis of the cell cycle and the expression of pluripotency and differentiation markers by RT-qPCR. Finally, the selected clone gave rise to the ESC line referred to as eGFP-NANOG.

### Cell cycle analysis for eGFP-NANOG ESC line validation

Cell cycle was analyzed by flow cytometry as previously described (Waisman et al., 2017). Briefly, single cell suspensions were fixed in 70% ethanol, rehydrated in PBS, stained with 25 μg/mL Propidium Iodide (Sigma) and incubated for 30 min. Then, samples were analyzed in a FACS Aria II flow cytometer (BD Biosciences). Data was compiled using Floreada software.

### Cell cycle classification with PCNA-RFP

Cells expressing the proliferating cell nuclear antigen (PCNA) fused to the red fluorescent protein (PCNA-RFP) were classified according to their PCNA nuclear distribution similarly to previous works in the field (Leonhardt et al., 2000; Sporbert et al., 2002; Pomerening et al., 2008; Leung et al., 2011; Barr et al., 2016; Wilson et al., 2016; Dutta et al., 2019; Velasquez et al., 2019; Velasquez et al., 2022; Xie and Bankaitis, 2022). Specifically, cells in early-S (E-S) phase present multiple small foci that distribute homogeneously within the nuclear space; mid-S (M-S) cells present foci close to the nucleoli or at the nuclear periphery; late-S (L-S) phase have fewer and bigger foci of the fusion protein and finally, cells in G1 and G2 have a homogeneous distribution (Supplementary Figure S1A). Those cells that did not show these characteristic PCNA features were discarded from further analyses.

Some previous works described methods based on machine learning to quantify PCNA distribution (Schonenberger et al., 2015) and we have also developed an image-based routine with this purpose (Presentation 2, Supplementary Materials and Methods). However, the performance of these methods is not better than that of the manual classification widely used in the literature (Leonhardt et al., 2000; Sporbert et al., 2002; Pomerening et al., 2008; Leung et al., 2011; Barr et al., 2016; Wilson et al., 2016; Dutta et al., 2019; Velasquez et al., 2019; Velasquez et al., 2022; Xie and Bankaitis, 2022) which constitutes the gold standard method to date.

S cells transitioning between phases usually present main features of a given phase combined with some features of a previous or consecutive phase. In these few cases, we used the following criteria for splitting cells between contiguous phases:

1. E-S to M-S transitioning cells: they show a combination of foci in the periphery (characteristic of M-S) and other in the nucleus interior (as observed in E-S). The cell is considered E-S or M-S if most of the foci are localized in the interior or the periphery, respectively. The cell is discarded from the analysis if the number of foci in both locations is similar.

2. M-S to L-S transitioning cells: they show a combination of foci in the periphery (characteristic of M-S) with bigger foci (as observed in L-S). We consider the cell as L-S if the cell shows few foci (although most of them are at the periphery) but bigger than the average size, otherwise, we classified the cell as M-S.

Miss-classifications between continuous phases result in smaller differences between the parameters calculated for these phases, thus the differences between these populations might be larger than those detected in this work.

### Identification of the different stages of S-phase by quantification of DAPI staining

Cells were plated on coated coverslips and transfected with a vector encoding PCNA-RFP as described above. Then, the samples were fixed with 4% PFA for 15 min, permeabilized with 0.1% Triton X-100 in PBS and stained with DAPI.

The cells were observed in a widefield fluorescence microscope set to collect DAPI fluorescence using a 10x air objective (NA = 0.3). We used the StarDist ImageJ plugin (Weigert et al., 2020) to segment all nuclei and quantify their DAPI integrated intensity. The integrated intensity of each cell was normalized to that of the mean of its colony (I_DAPI_, _normalized_) for correcting small variations of the illumination of the sample and/or in DAPI staining throughout the coverslip.

The same cell colonies were then observed by confocal microscopy to locate those transfected cells and collect PCNA-RFP images with high spatial resolution (60x oil objective, NA = 1.35). By following this procedure, we collected DAPI and PCNA images of the same cells in the widefield and confocal microscopes, respectively.

### RT-qPCR

RT-qPCR was performed and analyzed as previously described (Waisman et al., 2017; Verneri et al., 2020). Briefly, total RNA was extracted with QuickZol (Kalium Technologies). Then, RevertAid Reverse Transcriptase (ThermoFisher) was used to reverse transcribed RNA. cDNA Quantitative Real time PCR amplification was carried out using FastStart SYBR Green Master (Roche) in a LineGene 9600 engine (BioER). 2 or 3 biological replicates were performed in all the experiments, with 2 technical replicates for each condition. Gene expression was normalized to the geometrical mean of Gapdh values. The list of primers is included in Supplementary Table S1.

### Confocal microscopy

Confocal microscopy experiments were run in two FV1000 microscopes (Olympus) with either spectral or filter-based detectors. We checked a similar performance of these microscopes after running identical imaging and FCS experiments in both setups.

YPet and eGFP fusion proteins were visualized using a multi-line Ar laser tuned at 488 nm as excitation source whereas a 543 nm He-Ne laser was used for RFP fusion proteins. The laser light was reflected with a dichroic mirror DM405/488/543/635 for dual-color experiments and focused through an Olympus UPLSAPO 60X oil immersion objective (NA = 1.35) into the sample.

For confocal imaging, fluorescence was split into two channels set to collect in sequential mode between 500–530 nm and 580–680 nm (spectral detectors; measurements of YPet-OCT4, YPet-SOX2 and HP1α-eGFP in undifferentiated ESCs) or between 505–525 nm and 572–642 nm (emissions filters; measurements of YPet-OCT4, YPet-SOX2 and HP1α-eGFP in induced cells). A stack of 5 images was collected in each field and its average was used in further quantitative analyses.

Single-point FCS experiments were run using a laser power of ∼1 µW and collecting fluorescence in the range 500–530 nm (spectral detector; measurements of YPet-OCT4, YPet-SOX2 and HP1α-eGFP in undifferentiated ESCs) or 505–525 nm (emission filter; measurements of eGFP-NANOG undifferentiated ESCs and YPet-OCT4, YPet-SOX2 and eGFP-NANOG in induced cells) with the detectors set in the pseudo photon-counting mode.

We only run a single measurement (imaging and FCS experiments) in each cell to minimize photobleaching and photodamage that could also produce alterations in the cell cycle (Magidson and Khodjakov, 2013).

### Fluorescence correlation spectroscopy

The microscope was set to collect intensity in a single-point of the cell nucleus at 50,000 Hz during 2.7 min (OCT4 and SOX2 experiments) or at 25,000 Hz during 5.4 min (NANOG experiments). We only run a single experiment in each cell to minimize photodamage.

The auto-correlation function (ACF) data was calculated using the SimFCS program (LFD, Irvine, CA, United States) and fitted with Eq. 1 that considers the diffusion of the TFs and their binding to two populations of fixed sites (White et al., 2016):

$$G\left(\tau \right)=\frac{1}{2^{3/2}N}\left[f_{D}{\left(1+\frac{\tau }{\tau_{D}}\right)}^{-1}{\left(1+\frac{\tau }{{\omega^{2}\tau }_{D}}\right)}^{-1/2}+f_{short}e^{\frac{-\tau }{\tau_{short}}}+f_{long}e^{\frac{-\tau }{\tau_{long}}}\right]$$ (1)

where N is the mean number of fluorescent molecules in the confocal volume, τ_D_ is the characteristic diffusion time, ω is the ratio between axial and radial waists of the observation volume, and f_D_ is the freely diffusing population fraction. f_short_ and f_long_ are the population fractions bound to short-lived and long-lived targets, and τ_short_ and τ_long_ are their residence times, respectively. The reciprocal of the residence time corresponds to the dissociation constant k_off_.

### Image analyses

Images were analyzed as previously described (Verneri et al., 2020). Briefly, the coefficient of variation (CV) was calculated as the ratio between the standard deviation of the intensity and the mean intensity of the nucleus, both calculated excluding nucleoli. Foci were identified in binarized images of nuclei considering an intensity threshold of mean + 2 × Standard deviation. The number and mean intensity of these foci were then calculated using the ImageJ plugin “Analyze Particles.” We only considered those structures with sizes ≥ optical resolution.

### Statistical analysis

Results from microscopy experiments were expressed as mean ± SEM of at least three biological replicates. Statistical significance between groups was analyzed using Linear Mixed Models. Residuals fitted normal distribution and homogeneity of variance. Each cell cycle phase was compared with its previous phase. Differences were considered as significant at *p*-value ≤ 0.05. Statistical analysis was performed using the gls package of RStudio.

## Results

### SOX2 and OCT4 condensates reorganize during the early- to mid- S phase transition

The core pluripotency TFs present a heterogeneous distribution in the nucleus with few foci (Verneri et al., 2020) that, at least in the case of OCT4, seem to play a relevant role in chromatin organization during interphase (Wang et al., 2021). Here, we use the term *condensates* to refer to as these membrane-less foci without implications on the molecular mechanism driving the formation of these structures.

We first asked whether the distribution of SOX2 and OCT4, and particularly their condensates, change during S phase. With this aim, we used the ESC lines YPet-OCT4 and YPet-SOX2 which express either OCT4 or SOX2 fused to the fluorescent protein YPet upon induction by Dox (Verneri et al., 2020).

To identify ESCs in S phase, we transfected the cells with a vector that encodes the proliferating cell nuclear antigen (PCNA) fused to the red fluorescent protein (PCNA-RFP). This protein associates to the replication fork (Moldovan et al., 2007; Boehm et al., 2016) and it has been extensively used in the literature to identify cells in S phase (Pomerening et al., 2008; Barr et al., 2016; Zerjatke et al., 2017; Grant et al., 2018). PCNA changes its nuclear distribution during the different stages of S phase (Leonhardt et al., 2000; Somanathan et al., 2001; Sporbert et al., 2002; Essers et al., 2005; Held et al., 2010; Schonenberger et al., 2015) and thus it has been used as a reporter of these different stages in both fixed (Dutta et al., 2019; Velasquez et al., 2019; Lin et al., 2020; Velasquez et al., 2022; Xie and Bankaitis, 2022) and living cells (Ersoy et al., 2009; Piwko et al., 2010; Leung et al., 2011; Wilson et al., 2016).

**FIGURE 1:**
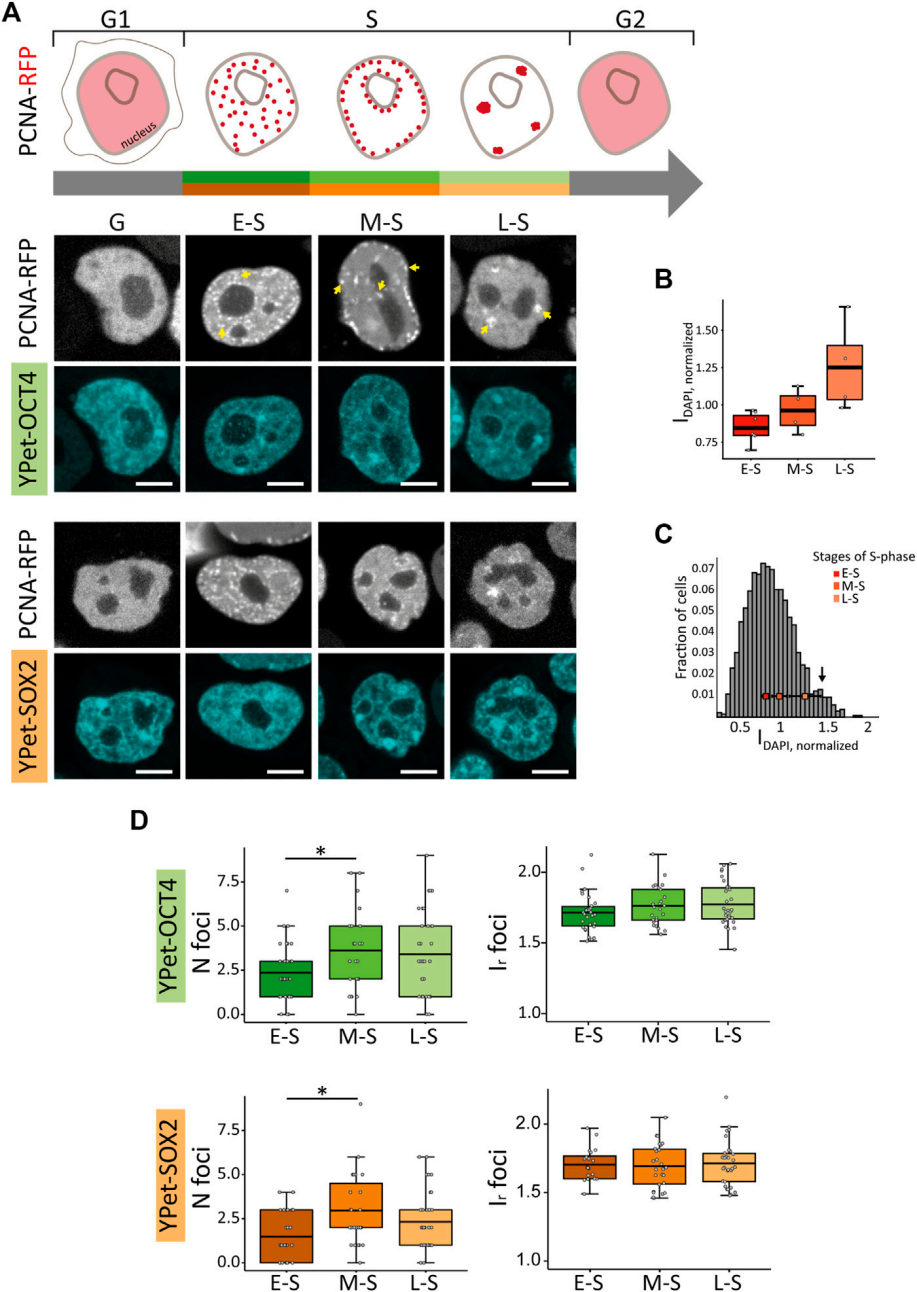
PCNA, SOX2 and OCT4 distribution in undifferentiated ESCs in G, E-S, M-S or L-S phases. **(A)** The cartoon represents PCNA distribution in these phases. Representative images of YPet-OCT4 and YPet-SOX2 ESCs transfected with a vector encoding PCNA-RFP in the different phases (G: G1 and G2). Arrows point to the characteristic foci observed in each phase. Scale bar: 5 µm. **(B)** DAPI normalized integrated intensity (I_DAPI, normalized_) for the E-S, M-S and L-S cells (dark to light red) classified according to PCNA distribution represented in boxplots; the thick, black lines represent mean values. **(C)** Intensity distribution of DAPI in ESCs (n cells = 1,362). The peak at 1.4 (arrow) probably corresponds to G2 cells and consequently, the region of the histogram with lower intensities than those of E-S cells probably includes cells in G1. The mean I_DAPI, normalized_ ± standard error determined in E-S, M-S and L-S cells (dark to light red) are plotted overlapped to the histogram. **(D)** Mean values of number of foci per cell (N foci, left) and relative foci intensity (I_r_ foci, right) for YPet-OCT4 (top, green) and YPet-SOX2 (bottom, orange) determined in E-S, M-S and L-S cells (dark to light). The thick, black lines in boxplots represent the mean values. *p-value < 0.05. Number of analyzed cells: YPet-OCT4: 34 (E-S), 26 (M-S), 33 (L-S); YPet-SOX2: 30 (E-S), 27 (M-S), 35 (L-S).

In line with these previous data, we observed that cells in early-S (E-S) phase showed multiple small foci of PCNA-RFP that distributed homogeneously within the nuclear space; mid-S (M-S) cells presented foci close to the nucleoli or at the nuclear periphery whereas cells transiting late-S (L-S) phase showed fewer and bigger foci of the fusion protein (Figure 1A; Supplementary Figure S1A). As expected, E-S, M-S and L-S cells presented increasing levels of DAPI intensities (Figures 1B, C) confirming that PCNA distribution allows the identification of cells in different stages of S-phase. Furthermore, the area of the optical section of nuclei in M-S and L-S phases was significantly larger than those of E-S cells (Supplementary Figure S1B), as expected from the increase of the nuclear volume during DNA replication (Maeshima et al., 2011). Finally, cells in G1 and G2 (herein referred to as G cells) showed a homogeneous distribution of PCNA-RFP (Figure 1A; Supplementary Figure S1A). Cells in mitosis were identified by the recruitment of the pluripotent TFs to the condensed chromosomes (Supplementary Figure S2A) as previously reported (Deluz et al., 2016), and were not included in further analyses.

We should also mention that these single-cell microscopy observations require cells growing attached to a substrate and thus we could not use cell sorting to select cells in specific phases of the cell cycle. The attachment of cells to the substrate takes hours and, during this period, the cell cycle of the sorted cells progress and the population become more heterogeneous.

Next, we quantified the distributions of YPet-OCT4 and YPet-SOX2 in single, live cells by calculating the coefficient of variation (CV), that provides information of the overall distribution of the fluorescent TFs, the mean number of foci (N_foci_) and their intensity relative to that of the nucleus (I_r,foci_). These parameters were previously used to obtain a quantitative description of the heterogeneous distribution of nuclear proteins including TFs such as OCT4 and SOX2 (Htun et al., 1999; Wu et al., 2006; Stortz et al., 2020; Verneri et al., 2020).

Although these parameters were not significantly different between G and S cells (Supplementary Figures S2B, C), changes were evident during the different stages of S phase (Figure 1D; Supplementary Figures S2D). Specifically, the number of foci in YPet-OCT4 (N cells: 34, 26 and 33 for E-S, M-S and L-S, respectively) and YPet-SOX2 (N cells: 30, 27, 35 for E-S, M-S and L-S, respectively) cells significantly increased after the E-S to M-S phase transition (Figure 1D). This observation could be related to the early timing of replication of active genes (Rhind and Gilbert, 2013; Hiratani and Takahashi, 2019); we hypothesize that pluripotency-associated OCT4 and SOX2 target genes probably replicate during E-S phase in PSCs and thus, those pluripotency TFs molecules that detach from replicating DNA might be further recruited to condensates.

In a previous work (Verneri et al., 2020), we showed that OCT4 and SOX2 condensates colocalize with regions of high chromatin compaction in ESCs, as evidenced by fluorescent versions of the histone H2B and the heterochromatin protein 1 (HP1α), a protein commonly associated with silenced heterochromatin regions (Grewal and Jia, 2007). Therefore, we next asked if the formation of new TF condensates after the E-S to M-S transition occurs concomitantly to the formation of compacted chromatin domains.

### HP1α foci reorganize at later stages of S phase

**FIGURE 2:**
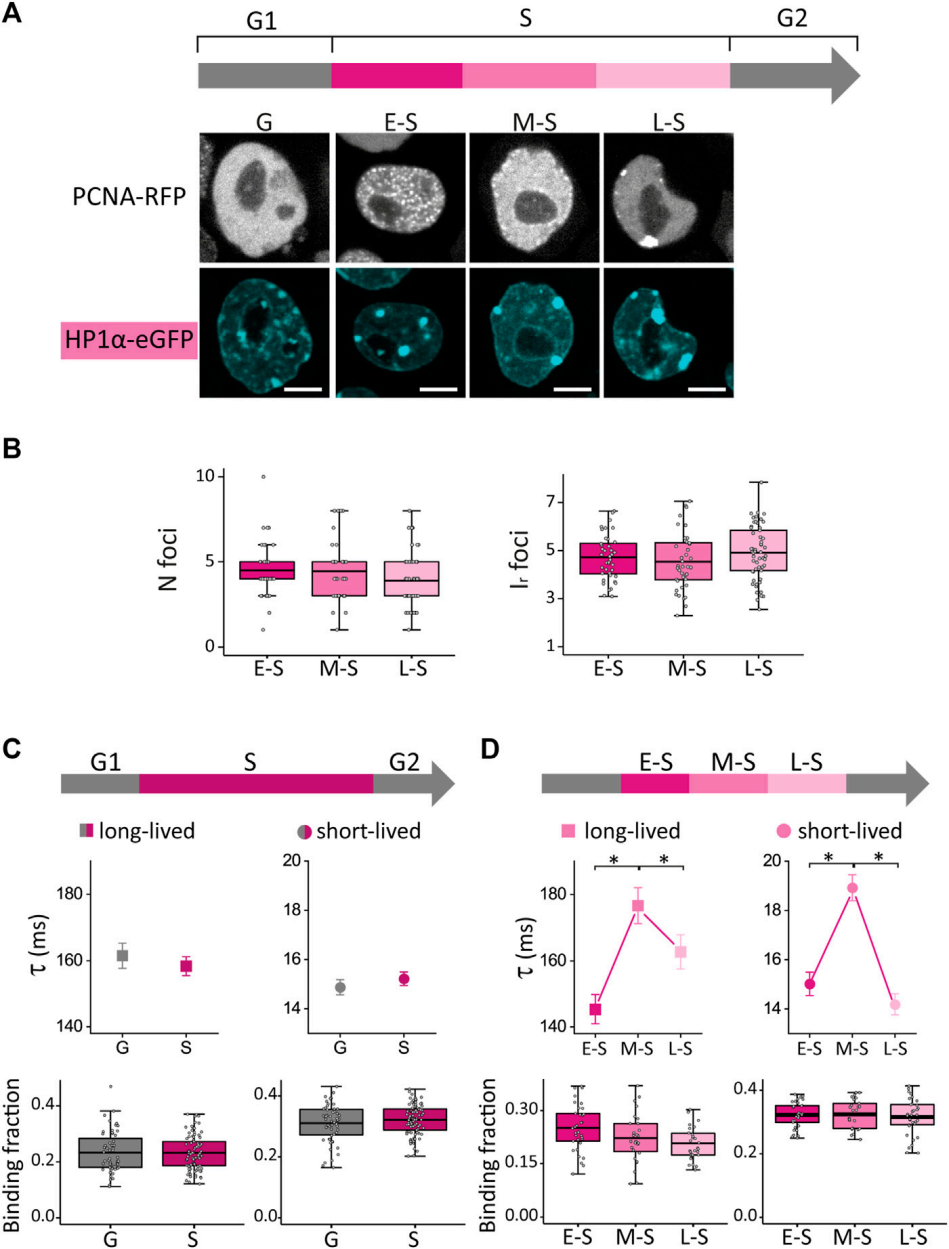
HP1α distribution and dynamics in undifferentiated ESCs in G, E-S, M-S or L-S phases. **(A)** ESCs were co-transfected with PCNA-RFP and HP1α-eGFP. Representative images of cells in G, E-S, M-S or L-S. Scale bar: 5 µm. **(B)** Mean values of N foci (left) and I_r_ foci (right) for HP1α-eGFP in cells in E-S, M-S and L-S (dark to light) phases. **(C)** HP1α-eGFP long-lived (left, squares) and short-lived (right, circles) binding times and the corresponding fractions were obtained from ACF curves during G (gray) and S (pink) phases or **(D)** during the different stages of S phase: E-S, M-S and L-S (dark to light pink). The thick, black lines in boxplots represent the mean values. *p-value < 0.05. Number of analyzed cells: 40 (E-S), 41 (M-S), and 57 (L-S); and FCS data: 58 (G), 35 (E-S), 27 (M-S) and 33 (L-S).

We analyzed the distribution of HP1α in ESCs co-transfected with vectors encoding HP1α fused to the enhanced green fluorescent protein (HP1α-eGFP) and PCNA-RFP by following the procedures described above. In line with previous results (Dialynas et al., 2007; Hinde et al., 2015; Strom et al., 2017), HP1α-eGFP formed foci in the nucleus of these ESCs (Figure 2A).

We first compared S and G cells and found that both CV and I_r,foci_ were significantly higher in S phase suggesting that HP1α is recruited to foci during DNA replication (Supplementary Figure S3A). Next, we analyzed HP1α distribution during S phase (Figure 2B; N cells: 40, 41 and 57 for E-S, M-S and L-S, respectively) and observed an increase of the CV value after the M-S to L-S transition (Supplementary Figure S3B) indicating that this transition promotes a more heterogeneous distribution of this protein.

Previous works showed that gene-poor, HP1-associated heterochromatin replicates at L-S phase (Ryba et al., 2010; Julienne et al., 2013; Rhind and Gilbert, 2013; Wootton and Soutoglou, 2021) and thus, we speculate that those changes in HP1α distribution occurring after the M-S to L-S transition could be due to a reorganization of HP1α required for heterochromatin duplication.

We should emphasize that the parameters characterizing the distribution of HP1α were not significantly different in E-S and M-S cells (Figure 2B; Supplementary Figure S3B), indicating that those changes of OCT4 and SOX2 distributions observed after this transition (Figure 1D) cannot be directly attributed to a reorganization of chromatin structure sensed through HP1α distribution.

To get further insights into the reorganization of HP1α occurring through the cell cycle, we used fluorescence correlation spectroscopy (FCS), a technique that provides exquisite information on the dynamical distribution and interactions of nuclear proteins in cells and whole organisms (Clark et al., 2016; White et al., 2016; Stortz et al., 2017; Cosentino et al., 2019; Verneri et al., 2020). Supplementary Figure S3C shows the ACF data obtained at different stages of the cell cycle (N cells: 58, 35, 27 and 33 for G, E-S, M-S and L-S, respectively). In line with our previous work (Cosentino et al., 2019), we fitted these data with Eq. 1, derived from a model that considers the diffusion of this protein and its interactions with chromatin targets in two distinct temporal windows (White et al., 2016; Stortz et al., 2017; Verneri et al., 2020).

Whereas HP1α-eGFP presented similar dynamics in S and G cells, we detected an increase of the lifetimes of HP1α-chromatin interactions in M-S cells (Figures 2C, D). A previous work (Quivy et al., 2008) showed that, to progress through M-S and L-S stages and to achieve the replication of pericentric heterochromatin, HP1 must interact with the chromatin assembly factor 1 complex (CAF-1), which is fundamental for heterochromatin organization in ESCs (Houlard et al., 2006). Therefore, we speculate that the changes detected in M-S cells could be indirectly related to the required establishment of interactions between HP1 and CAF-1 for heterochromatin replication in later stages of S phase; further work needs to be done to assess this hypothesis. Relevantly, a previous work also reported a distinct distribution of these molecules in mid-late S cells in which CAF-1, together with newly synthesized DNA, concentrates at the periphery of pericentric heterochromatin domains (Quivy et al., 2004).

We also analyzed publicly available data from genome-wide ATAC-seq experiments to get insights in the chromatin accessibility of ESCs (Friman et al., 2019) and observed that chromatin accessibility is constantly changing during the cell-cycle (Supplementary Figure S4). Particularly, heatmap analyses of chromatin accessibility changes reveal important variations in both late G1 and S phases. We should highlight that these results provide a broad panorama of the accessibility changes and cannot be directly compared to or associated with our experimental results since cells in Friman et al. (2019) were classified as early G1, late G1, S and S + G2.

Taken together, our results show that HP1α distribution and dynamics change during S phase. Relevantly, these modifications are not parallel to those observed for the pluripotent TFs (Figure 1) indicating that other, yet unknown factors different from chromatin compaction define the redistribution of OCT4 and SOX2 observed during S phase in ESCs.

### The pluripotency TFs OCT4 and SOX2 display different dynamics during S phase

To get insights into the dynamical organization of OCT4 and SOX2 during S phase, we run FCS experiments in YPet-OCT4 and YPet-SOX2 cells also transfected with the PCNA-RFP vector. We have previously shown that this technique allows detecting changes in the dynamical organization of pluripotency TFs occurring at early stages of differentiation (Verneri et al., 2020) and those indirectly produced by a histone acetyltransferase that modify the compaction of chromatin (Cosentino et al., 2019).

**FIGURE 3:**
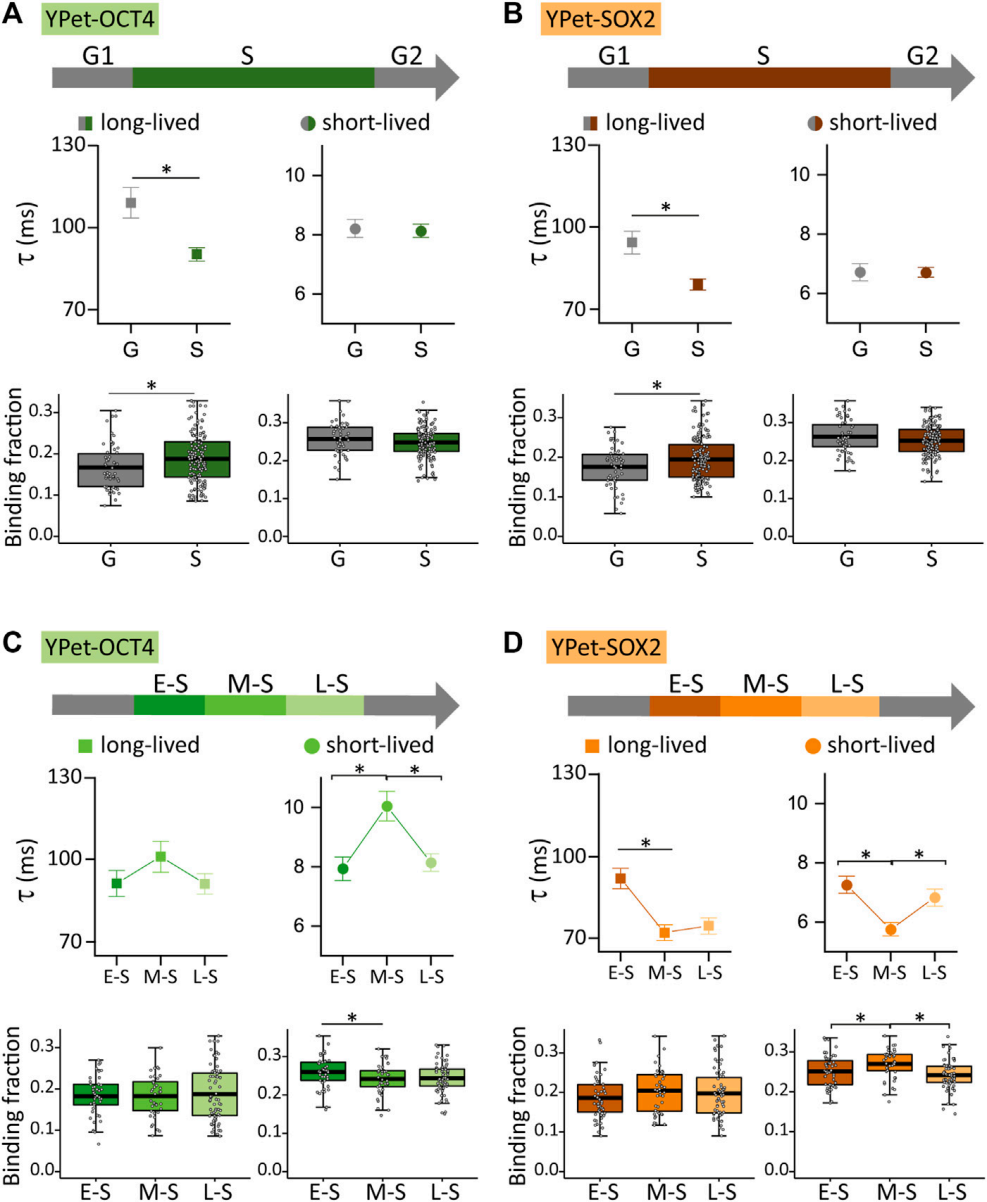
YPet-OCT4 and YPet-SOX2 dynamics during the cell cycle of ESCs. **(A)** YPet-OCT4 and **(B)** YPet-SOX2 long-lived (left, squares) and short-lived (right, circles) binding times (top) and the corresponding fractions (bottom) obtained from ACF curves during G (gray) or S (color) phases. **(C)** YPet-OCT4 and **(D)** YPet-SOX2 long-lived (left, squares) and short-lived (right, circles) binding times (top) and the corresponding fractions (bottom) obtained from ACF curves during E-S, M-S or L-S (dark to light) phases. The thick, black lines in boxplots represent the mean values. *p-value < 0.05. Number of FCS data for YPet-OCT4: 53 (G), 47 (E-S), 42 (M-S) and 68 (L-S); and for YPet-SOX2: 56 (G), 60 (E-S), 46 (M-S) and 63 (L-S).

Figures 3A, B show that the dynamics of both TFs was significantly different in S and G cells (N cells OCT4: 53 and 157 for G and S; N cells SOX2: 56 and 169 for G and S, respectively). Specifically, S cells showed a higher proportion of TF molecules attached to long-lived chromatin targets, but these interactions were faster than those observed in G cells. These observations suggest that OCT4 and SOX2 reorganize among chromatin targets during S phase.

FCS experiments also revealed subtle, albeit statistically different modifications in the dynamics of OCT4 (N cells: 47, 42 and 68 for E-S, M-S and L-S, respectively) and SOX2 (N cells: 60, 46 and 63 for E-S, M-S and L-S, respectively) during S phase. Particularly, OCT4 detached from chromatin targets after the E-S to M-S phase transition and those bound OCT4 molecules remained longer at short-lived targets (Figure 3C). On the other hand, SOX2 redistributed to more labile chromatin targets in M-S cells as the lifetime of SOX2-chromatin interactions decreased and its short-lived bound fraction increased after this transition (Figure 3D). These observations indicate that the E-S to M-S transition involves changes of TF-chromatin interactions that depend on the TF identity concomitant with a reorganization of TFs condensates (Figure 1). Figures 3C, D also show changes in the lifetimes of short-lived interactions of both TFs and a reduction in the fraction of SOX2 molecules bound to the short-lived targets during the M-S to L-S transition.

Taken together, our results show that the landscape of interactions of OCT4 and SOX2 with chromatin targets changes during the DNA replication. Although these core pluripotency TFs also act as heterodimers in the regulation of many genes (Remenyi et al., 2003; Boyer et al., 2005; Chew et al., 2005; Rodda et al., 2005), the different dynamics during S phase is consistent with their different chromatin-binding landscape (Rizzino, 2013) and interactions with other proteins (Buecker et al., 2014).

### The distribution and dynamics of SOX2 and OCT4 in E-S cells respond to differentiation cues received in the preceding G1 phase

We mentioned before evidence showing that mouse ESCs respond to differentiation cues after cell division (Chaigne et al., 2020) and specifically, in G1 phase (Waisman et al., 2017). Also, we have previously reported that differentiation stimuli trigger early changes in the distribution and dynamics of SOX2 and OCT4 that precede their downregulation (Verneri et al., 2020). In this context, we next asked if differentiation cues received in G1 affect the distribution and/or dynamics of these pluripotency TFs in the subsequent E-S phase.

**FIGURE 4:**
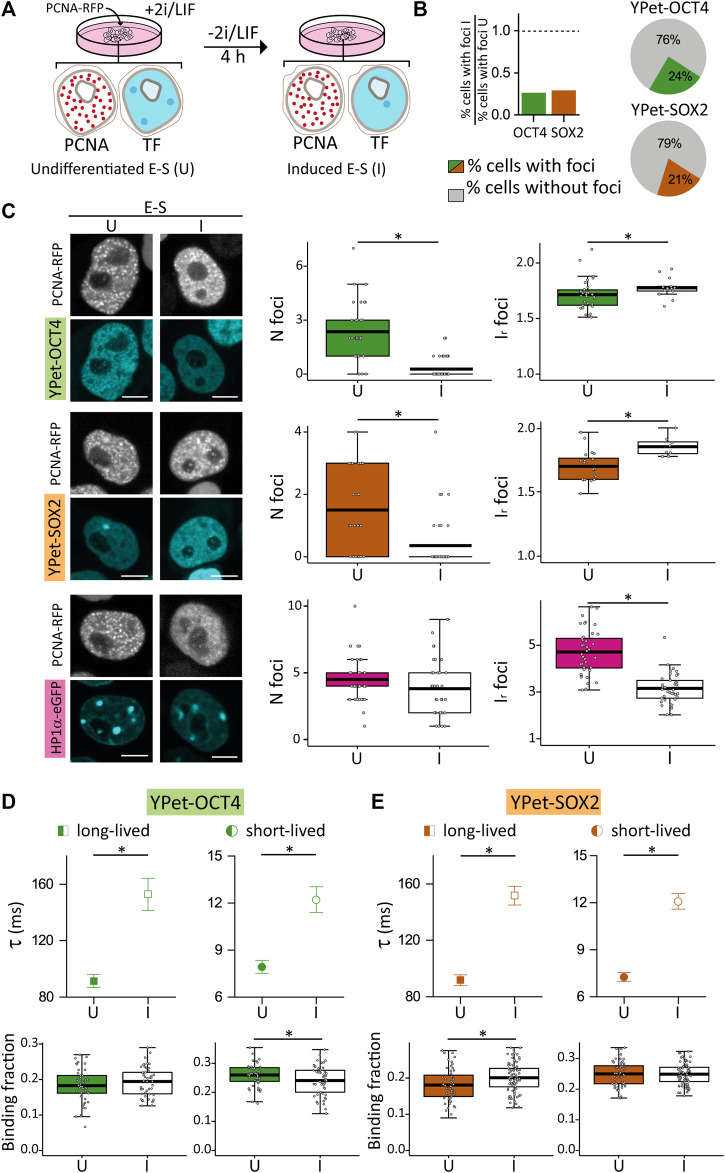
Distribution and dynamics of HP1α-eGFP, YPet-OCT4 and YPet-SOX2 in undifferentiated E-S cells or after 4 h of induction of differentiation. **(A)** Cartoon schematizing the experimental procedure. **(B)** Proportion of cells with at least one focus in induced E-S cells (I) relative to that of undifferentiated E-S cells (U). Pie charts showing the proportion of induced E-S cells without foci (gray) or with at least one focus (YPet-OCT4 or YPet-SOX, green and orange, respectively). **(C)** Representative images of undifferentiated and induced E-S cells expressing YPet-OCT4 (top), YPet-SOX2 (middle) and HP1α-eGFP (bottom) and mean values of N foci (left) and I_r_ foci (right) (filled and empty symbols for U and I cells, respectively). Scale bar: 5 µm. **(D,E)** long-lived (squares) and short-lived (circles) binding times and the corresponding fractions obtained from ACF data collected in undifferentiated (filled symbols) or induced (empty symbols) E-S cells expressing **(D)** YPet-OCT4 or **(E)** YPet-SOX2. The thick, black lines in boxplots represent the mean values. *p-value < 0.05. Number of induced E-S cells analyzed: 58 (YPet-OCT4), 39 (YPet-SOX2), 37 (HP1α-eGFP); and FCS data: 52 (YPet-OCT4), 77 (YPet-SOX2).

We used YPet-OCT4 and YPet-SOX2 ESCs expressing PCNA-RFP and induced differentiation by LIF/2i withdrawal (Verneri et al., 2020) during 4 h (Figure 4A). G1 lasts approximately 2–3 h (Waisman et al., 2017) and thus E-S cells observed after the treatment most likely received the differentiation signal in the preceding G1 phase.

Surprisingly, most E-S cells did not show TF condensates after differentiation induction (Figure 4B); indeed, only 24% of YPet-OCT4 (N cells: 58) and 21% of YPet-SOX2 (N cells: 39) E-S cells that received the differentiation signal (herein referred to as induced E-S cells) presented the characteristic TF foci. Consequently, N_foci_ was lower in induced E-S cells although the remaining foci were brighter than those observed in undifferentiated E-S cells (Figure 4C) suggesting a higher recruitment of TF molecules to these fewer foci. Also, induced E-S cells have higher CV (Supplementary Figures S5A, B) suggesting an overall, more heterogeneous distribution of OCT4 and SOX2 in the nucleus of these cells compared to undifferentiated E-S cells.

To explore if these changes in pluripotency TF condensates are related to evident modifications of chromatin condensation in this early time-window of differentiation, we analyzed HP1α-eGFP distribution in E-S cells exposed to the differentiation stimulus in G1 phase (N cells: 37). We detected HP1α foci in every induced E-S cell suggesting that the lower number of OCT4 and SOX2 condensates are not a direct consequence of a similar reorganization of HP1α. The intensity of HP1α foci as well as CV values were significantly lower than those observed in undifferentiated E-S cells indicating that the differentiation stimuli triggered a more homogeneous distribution of HP1α in E-S phase (Figure 4C; Supplementary Figure S5C). Although chromatin in pluripotent ESCs is globally decondensed and increases its compaction during differentiation (Azuara et al., 2006; Meshorer et al., 2006; Gokbuget and Blelloch, 2019), this last result could be interpreted considering that there is a short period of chromatin decondensation during the first stages of differentiation (Chalut et al., 2012; Petruk et al., 2017). Particularly, chromatin decondenses after 2–4 h of differentiation induction in mouse ESCs (Petruk et al., 2017). Despite the methodological differences, this previous work suggests that decondensation occurs in a temporal window similar to that analyzed in our experiments.

To get further insights into the reorganization of OCT4 and SOX2, we run FCS experiments similar to those described above (Figures 4D, E).

Figures 4D, E show that induced E-S cells presented significantly higher lifetimes of OCT4 (N cells: 52) and SOX2 (N cells: 77) interactions with chromatin in comparison to undifferentiated E-S cells. These results suggest that differentiation induction in G1 promotes stronger OCT4 and SOX2 interactions with chromatin in E-S cells. Also, these TFs reorganized among targets as revealed by the higher long-lived binding fraction of SOX2 and the smaller short-lived binding fraction of OCT4 determined in induced E-S cells.

### NANOG dynamics in early S cells respond to differentiation cues received in G1

In contrast to OCT4 and SOX2, NANOG is associated with a naïve pluripotency state (Martello and Smith, 2014), the induction of differentiation results in its fast downregulation (Kalkan et al., 2017; Waisman et al., 2017; Verneri et al., 2020) and its expression levels also diminish during mitosis (Liu et al., 2017). Therefore, we next explored NANOG dynamics during the cell cycle and asked if it also responds to differentiation cues received in G1 phase as SOX2 and OCT4.

We first generated an ESC line that stably expresses NANOG fused to eGFP (eGFP-NANOG) as described in Materials and methods. We verified eGFP-NANOG expression and observed that the cell and colony morphologies and the cell cycle were similar to those corresponding to the parental cell line (Supplementary Figure S6A–C). We also induced differentiation by LIF/2i withdrawal and analyzed gene expression. Supplementary Figure S6D shows that the naïve pluripotency markers Nanog, Esrrb and Klf4 are downregulated at early differentiation and that the marker of primed pluripotency Oct6, which is upregulated early during differentiation (Kalkan et al., 2017; Waisman et al., 2017; Waisman et al., 2019), is upregulated, confirming that the new cell line is capable to exit naïve pluripotency despite expressing the fusion protein eGFP-NANOG.

Then, we transfected these cells with PCNA-RFP and run FCS experiments to analyze the dynamics of NANOG during the cell cycle.

**FIGURE 5:**
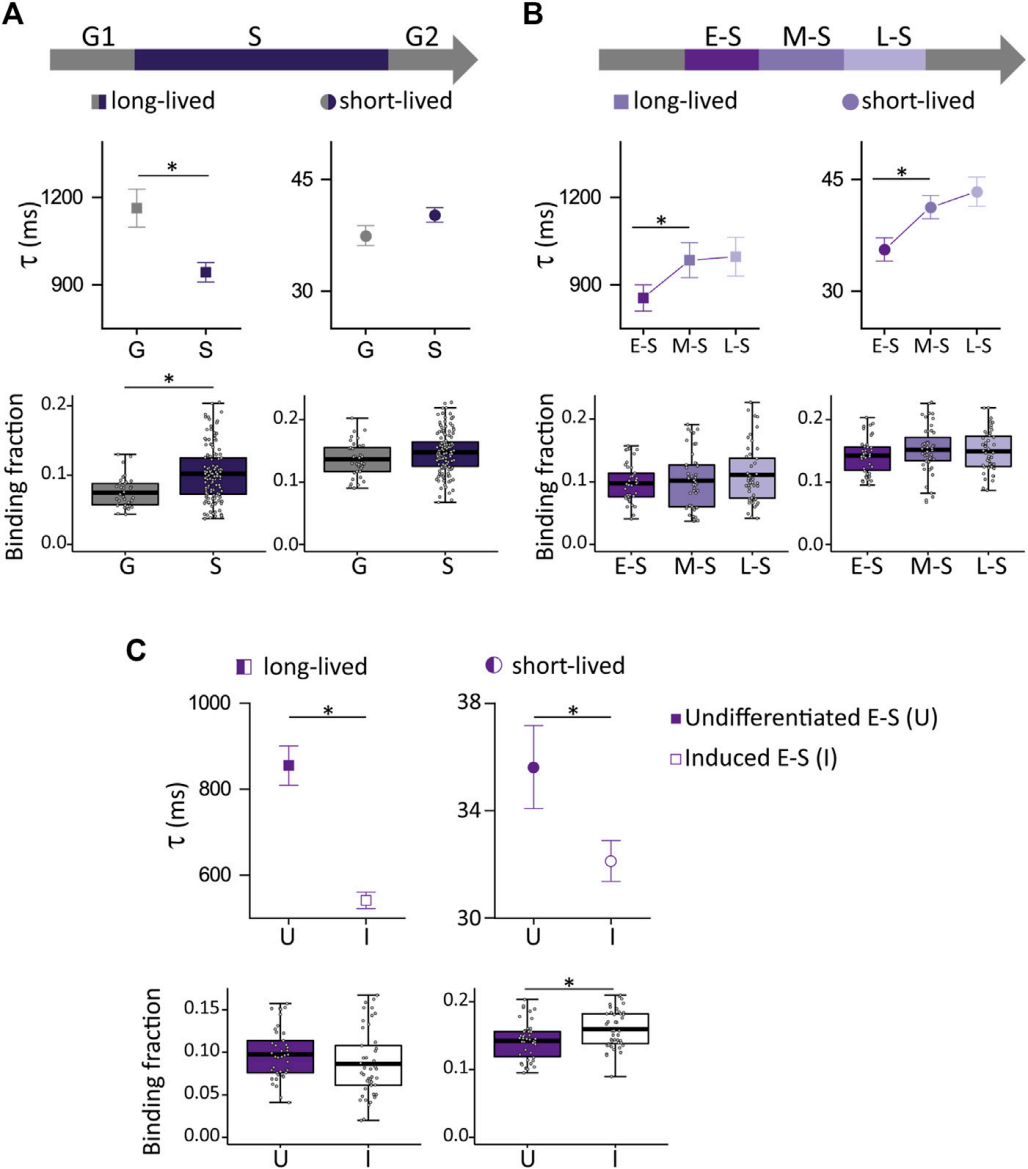
NANOG-GFP dynamics during the cell cycle of undifferentiated ESCs and in induced E-S cells. **(A,B)** NANOG-eGFP long-lived (left, squares) and short-lived (right, circles) binding times (top) and the corresponding fractions (bottom) obtained from ACF curves during **(A)** G (gray) or S (purple) phases or **(B)** during E-S, M-S or L-S (dark to light) phases. **(C)** long-lived (squares) and short-lived (circles) binding times and the corresponding fractions were obtained from ACF data collected in undifferentiated (filled symbols) or induced (empty symbols) E-S cells. The thick, black lines in boxplots represent the mean values. *p-value < 0.05. Number of FCS data: 41 (G), 43 (E-S), 47 (M-S), 69 (L-S), and 45 (induced E-S).

Figure 5A shows that the fraction of NANOG molecules in S cells involved in long-lived NANOG-chromatin interactions was higher while the lifetime of these interactions was lower in comparison to G cells (N cells: 41 and 156 for G and S, respectively), in line with the behavior observed for SOX2 and OCT4. Also, we studied the dynamics of NANOG during the different stages of S phase (N cells: 43, 47 and 69 for E-S, M-S and L-S, respectively) (Figure 5B) and found that the lifetime of NANOG-chromatin interactions increased after the E-S to M-S transition. These results show that NANOG also redistributes among chromatin targets during the cell cycle.

Next, we asked if NANOG dynamics in E-S phase responds to differentiation cues received in G1. Although Nanog expression levels decrease during differentiation, its protein levels do not change significantly after 6 h from naïve pluripotency exit (Yang et al., 2019). In line with this observation, RNA levels of Nanog are not significantly different after 5 h of receiving the differentiation stimuli in G1 (Waisman et al., 2017).

Surprisingly, NANOG behavior (Figure 5C) was almost the opposite of that observed for OCT4 and SOX2 (Figures 4D, E). Specifically, we found that the lifetimes of NANOG-chromatin interactions were significantly smaller in induced E-S cells (N cells: 45) while the short-lived fraction increased in comparison to undifferentiated E-S cells suggesting weaker NANOG-chromatin interactions in cells that were induced to differentiate in G1.

The different behavior between OCT4/SOX2 and NANOG could be related to the different functional roles of these pluripotency TFs discussed above. We hypothesize that differentiation cues trigger a redistribution of OCT4 and SOX2 among binding sites in the chromatin that might be required to switch to a differentiation program in line with their expected activity as pioneer factors (Friman et al., 2019). In contrast, the detachment of NANOG from chromatin sites in induced E-S cells probably constitutes one of the initial steps toward its downregulation and the consequent rapid repression of pluripotency-related genes.

## Discussion

A challenging and yet elusive question in Cell Biology is how a cell conserves its gene expression program during the cell cycle and between cell divisions or modifies it after receiving specific cues.

DNA replication has been raised as a critical phase for cell fate decisions. This process comprises chromatin changes at very different scales ranging from the nanoscopic detachment of nucleosomes to the remodeling of large chromosomal regions. While the epigenome helps to preserve the memory of the transcriptional state, it is challenged during DNA replication (Stewart-Morgan et al., 2019). Moreover, those DNA-involving processes such as transcription, which includes a plethora of molecules that directly/indirectly bind to certain chromatin regions, must also reorganize during and after DNA replication.

This process does not occur in the whole genome simultaneously; indeed, different chromatin regions are duplicated in a strict spatiotemporal program (Ding et al., 2021) that depends on several factors including the spatial localization and internal organization of the specific genomic regions (Wootton and Soutoglou, 2021). Roughly, euchromatin, facultative chromatin and constitutive heterochromatin in mammalian cells are replicated in E-S, M-S, and L-S phases, respectively (Heinz et al., 2018).

In mouse ESCs, nascent chromatin is both inaccessible to TFs and transcriptionally silent; chromatin accessibility and RNA polymerase occupancy restore within 2 h of replication (Stewart-Morgan et al., 2019). Also, specific TFs seem to play a relevant role in the preservation of the epigenetic memory during DNA replication (Soufi and Dalton, 2016; Vanzan et al., 2021).

In this context, we reasoned that pluripotency TFs and specifically OCT4 and SOX2 with reported pioneer activities (Iwafuchi-Doi and Zaret, 2014; Jerabek et al., 2014; Soufi et al., 2015; King and Klose, 2017; Friman et al., 2019; Roberts et al., 2021) probably play fundamental and closely related roles during S-phase that include the modulation of chromatin accessibility and the regulation of pluripotency genes. Thus, the propagation or switching between specific gene expression programs likely require these closely-related activities occurring in a specific and probably overlapped spatial and temporal sequence.

Our study revealed that the nuclear distribution of OCT4 and SOX2 changes during S phase in ESCs. First, the number of TFs condensates increased after the E-S to M-S phase transition. Relevantly, we did not detect a parallel modification of HP1α foci suggesting that the reorganization of OCT4 and SOX2 could not be explained exclusively by modifications of heterochromatic regions during S-phase.

Previous evidence suggests that OCT4 forms condensates with Mediator through a liquid-liquid phase separation process (Boija et al., 2018). On the other hand, Mediator condensates are proposed to include super-enhancers and contribute to gene expression regulation (Sabari et al., 2018). In addition, the core pluripotency TFs bind to super-enhancers that regulate cell fate commitment genes in stem cells (Hnisz et al., 2013; Whyte et al., 2013). Further works showed that OCT4 condensates play a key role in TAD reorganization which correlates with the redistribution of super-enhancers during reprogramming (Wang et al., 2021). Altogether, this evidence provides possible links between condensates, chromatin remodeling and the function of super-enhancers in ESCs (Hnisz et al., 2017), and allows us to speculate that the remodeling of TF condensates at early stages of S phase may be associated with the reestablishment of super-enhancers. Relevantly, these structures recover their accessibility with a faster kinetics than other genomic features (Stewart-Morgan et al., 2019).

In this work, we also found different dynamics of pluripotency TFs in S and G phases. Specifically, FCS revealed a higher amount of TF molecules bound to long-lived chromatin sites in S phase although these interactions were faster than in G phase. This general rearrangement towards relatively weaker TF-chromatin interactions observed for OCT4, NANOG and SOX2 could be related to the overall reduction of the transcriptional activity during DNA replication (Stewart-Morgan et al., 2019).

FCS analysis also revealed that pluripotency TFs present distinct dynamics during the different stages of S phase highlighting their particularities as key players on defining chromatin accessibility and gene expression regulation. Whereas OCT4 and SOX2 bind as heterodimers to many of their targets (Chew et al., 2005; Rodda et al., 2005; Wang et al., 2007; Chen et al., 2008), they can differentially modulate chromatin accessibility (Friman et al., 2019) and gene expression (Thomson et al., 2011). Recently, Friman et al. (2019) showed that OCT4 and SOX2 operate as pioneer TFs in a largely independent manner even at co-occupied sites across the cell cycle. In line with these observations, our results suggest that OCT4 and SOX2 present distinct chromatin-binding landscapes during S phase.

Several studies raised G1 as the cell cycle phase in which pluripotent stem cells are receptive to differentiation cues (Sela et al., 2012; Coronado et al., 2013; Pauklin and Vallier, 2013). The massive chromatin remodeling produced during DNA replication constitutes a window of opportunity in which transcription-related molecules could gain access to other chromatin sites and reshape the gene expression profile towards that required for establishing a new cell identity. Our previous findings (Waisman et al., 2017) also support this statement since inhibition of DNA replication interferes with the expected modifications in gene expression triggered by differentiation stimuli.

We observed that E-S cells that received the differentiation cue in the preceding G1 phase present a more homogeneous distribution of HP1α with dimmer foci in comparison to undifferentiated E-S cells, suggesting a fast remodeling and decondensation of heterochromatic regions produced at the onset of the differentiation process. These observations agree with previous works reporting a brief period of chromatin decondensation during the initial stages of differentiation (Chalut et al., 2012; Petruk et al., 2017).

We have previously reported that OCT4 and SOX2 modify their nuclear organization during early stages of differentiation (<48 h) that precede their downregulation (Verneri et al., 2020). Here, we observed massive changes in the organization of these pluripotency TFs in E-S cells after only 4 h of differentiation induction in G1. Particularly, E-S cells that were exposed to the differentiation signal in G1 presented a significantly lower number of OCT4 and SOX2 condensates than non-stimulated E-S cells. Considering the functional link between condensates and pluripotency-related super-enhancers described above, this observation could be related to a remodeling and a consequent loss of activity of these super-enhancers during the exit of pluripotency.

Moreover, we determined higher lifetimes of OCT4 and SOX2 interactions with chromatin in induced E-S cells. We could speculate that these long-lasting interactions could be related to a redistribution of these TFs, associated with the remodeling of the chromatin landscape assessed through HP1α. Ιt remains elusive whether the remodeling is a consequence of the pioneer function of these TFs or if the changing chromatin configuration exposes new binding sites for these TFs. It would be interesting for further research to reveal the identity of the loci affected by the pluripotency TFs redistribution.

Importantly, we observed an almost opposed behavior for NANOG, consistent with its naïve pluripotency TF role and its early downregulation at the exit of pluripotency (Silva et al., 2009; Kalkan et al., 2017). Specifically, we detected faster NANOG-chromatin interactions in E-S cells exposed to the differentiation signal in G1, suggesting that this TF detaches from chromatin targets thus triggering the downregulation of pluripotency-related genes. To our knowledge, there are no reports of pioneer activity of NANOG in mammalian stem cells although a recent work identified NANOG as responsible for opening chromatin at high nucleosome affinity regions in the zebrafish embryos (Veil et al., 2019).

In this work we followed the standard criteria for classification of the cells widely used in this field (Leonhardt et al., 2000; Sporbert et al., 2002; Pomerening et al., 2008; Leung et al., 2011; Barr et al., 2016; Wilson et al., 2016; Dutta et al., 2019; Velasquez et al., 2019; Velasquez et al., 2022; Xie and Bankaitis, 2022). Although the different PCNA distributions at the different cell cycle stages are easily recognized by eye inspection, this classification has inherent observer errors. Unfortunately, there are only a few automated routines described in the literature to de automatic classification of PCNA labeled cells (Schonenberger et al., 2015) and they are based on machine learning to do fast processing of large data sets as those obtained in high throughput microscopy experiments. For the learning, all these algorithms require a large number of annotated data which are manually classified. Then, the overall criteria acquired during the learning depend on the manually-based classification of a data set similar, in size, to those obtained in our confocal experiments. Relevantly, the performance of the reported routines is lower than the manual classification (Schonenberger et al., 2015). To our knowledge, there are no other methods described in the literature that provide a clear advantage (in terms of precision) over the manual classification methodology widely used by the scientific community.

Taken together, our data revealed a continuous reorganization of the core pluripotency TFs in the nuclear space during S phase. Also, our findings suggest that E-S cells rapidly respond to differentiation stimuli in G1 by reorganizing HP1α-associated chromatin regions and dissolving SOX2 and OCT4 condensates. Moreover, our study highlights the different roles of the pluripotency TFs in this early response; whereas OCT4 and SOX2 increased their lifetimes of TF-chromatin interactions, NANOG showed impaired interactions with chromatin after differentiation induction probably as a rapid regulation mechanism that precedes its own downregulation and that of other pluripotency genes. Further work needs to be done to understand if NANOG detachment is related to the chromatin remodeling and the concomitant reorganization of the pioneer TFs, OCT4 and SOX2. It will be also interesting to study by ChIP-seq analysis the identity of those loci affected by the redistribution of pluripotency TFs during S-phase in undifferentiated and induced cells. Additionally, the use of high throughput microscopy methods with reduced light-exposure to minimize photodamage of the cells will allow observing single cells as they progress through S phase. These experiments could provide exquisite information on the time evolution of single cells also allowing the identification of behavioral heterogeneities.

## Data Availability

The raw data supporting the conclusion of this article will be made available by the authors, without undue reservation.

## Abbreviations

ACF, Autocorrelation function; CV, Coefficient of variation; ESC, Embryonic stem cell; E-S, Early-S; FCS, Fluorescence correlation spectroscopy; L-S, Late-S; M-S, Mid-S; PSC, Pluripotent stem cell; TAD, Topologically associating domains; TF, Transcription factor.
