# Supplementary material for: The dynamical organization of the core pluripotency transcription factors responds to differentiation cues in early S-phase
Source: Front Cell Dev Biol. 2023 May 4;11:1125015. doi: 10.3389/fcell.2023.1125015 (PMC10192714; doi:10.3389/fcell.2023.1125015)
Supplement: Supplementary file 1 [file DataSheet2.pdf]

1. Supplementary Materials and Methods

We developed a simple ImageJ routine to quantitatively classify PCNA distribution, based on identifying the features recognized by eye-inspection (**Figure I**). To optimize the algorithm, we used a set of ~20 images for each cell cycle phase which were classified by eye-inspection.

For these quantifications, we first generated binary images of the nuclei (**Figure I A**) considering an intensity threshold equal to the mean + 2 x Standard deviation and then quantified the following parameters in the binary images:

**Parameter**	**definition**
N_{Foci}	total number of foci
Mean Foci Area	average size of foci
$N_{Foci>0.3\ \mu m^2}$	number of foci with area > 0.3 μm^2
$N_{Foci\ Border}$	number of foci at the nucleus and nucleoli borders
$N_{Foci\ Center}$	number of foci in the nuclear interior
δ_{Border}	$N_{Foci\ Border}$ divided by the area of the border
δ_{Center}	$N_{Foci\ Center}$ divided by the area of the center

We used the *analyze particles* plugin to determine N_{Foci} and the area of these foci. We only considered those structures with sizes ≥ 0.067 μm^2 (i.e., the image area of subdiffraction structures). To quantify $N_{Foci\ Border}$, we detected the borders of the nuclei and nucleoli in binary images of the whole nuclei using the *outline* plugin. Then, we used the *variance* filter to make these regions thicker in order to include peripheral foci. $N_{Foci\ Center}$ was calculated using a binary image of the whole nuclei that exclude these borders (**Figure I A**).

Figure I B shows the routine workflow. In the first step of the routine, we split G and S cells using the parameter N_{Foci}. G cells were considered as those presenting less than one focus. S cells were further classified in consecutive steps of the routine.

Particularly, we considered the combined parameters ($N_{Foci\ i>0.3\ \mu m^2}/N_{Foci}$) and ($N_{Foci\ Border}/N_{Foci\ Center}$) to differentiate cells in different S stages. **Figure I B** shows that cells in the gray area correspond to E-S and M-S cells, whereas those included in the white region were L-S and M-S cells.

We further analyzed the combined E-S and M-S group (gray area) using parameters that inform on the distribution of the foci within the nucleus space. Particularly, we plotted N_{Border} vs. N_{Center}. Those cells in the light-pink and white areas correspond to M-S or E-S cells, respectively.

Finally, we analyzed the combined L-S and M-S group according to the size of the foci. Specifically, L-S cells were considered as those presenting foci sizes ≥ 0.3μm^2.

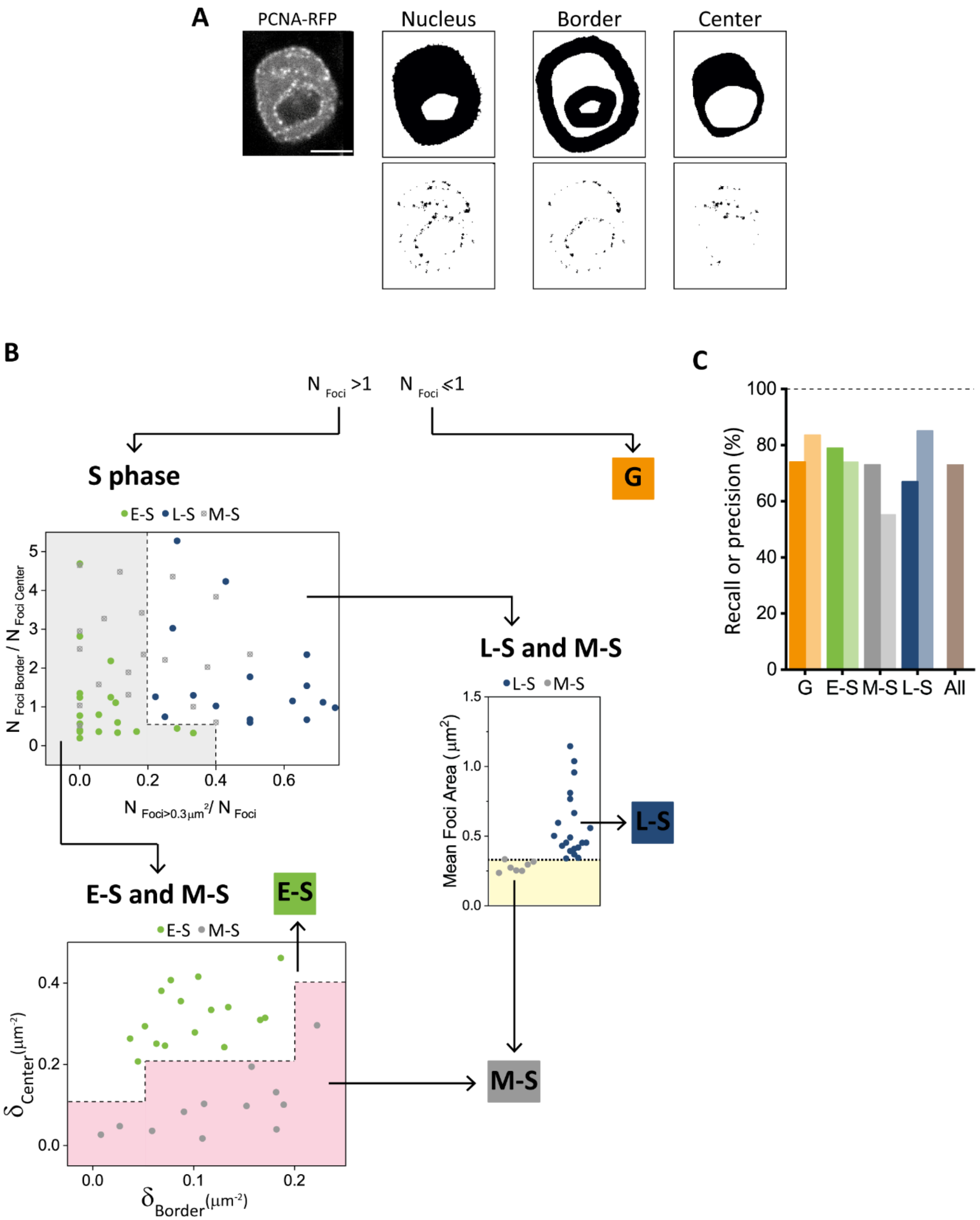

Figure I. (**A**) Representative image of a cell expressing PCNA-RFP, its binary images generated as described in the text. Scale bar: 5µm. (**B**) Scheme of the classification algorithm. Green, gray and blue dots correspond to cells manually classified as E-S, M-S and L-S, respectively. (**C**) Classification recall (dark colors) and precision (light colors) as defined in (Schonenberger et al., 2015) for G (orange), E-S (green), M-S (grey), L-S (blue) cells and all phases combined (brown).

Although this routine presents a better performance than previously methods (Schonenberger et al., 2015) (**Figure I C**), we observed many failures in the classification. When visually observing those cells that were incorrectly classified, we detected that the algorithm identifies some relatively more intense regions of the background as foci or on the contrary, it fails to identify some dimmer PCNA foci easily observed by eye (**Figure II**).

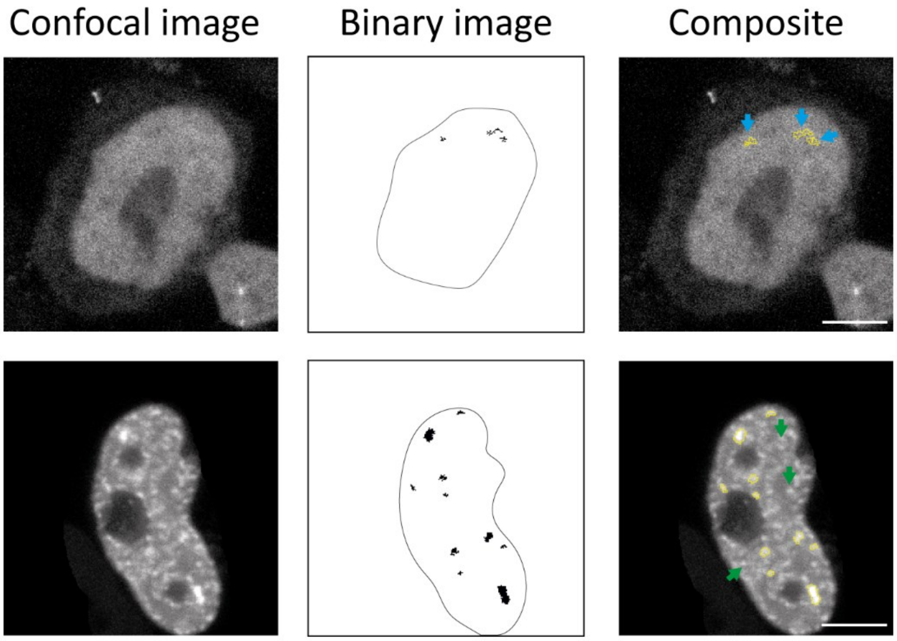

Figure II. Examples of incorrect classifications of cells. Representative PCNA and foci detected with the ImageJ routine (continuous lines are the outline of the nucleus) showing miss-identification of some relatively more intense regions of the background as foci (top, blue arrows) or failure to identify some foci (bottom, some examples are pointed with green arrows). Scale bars: 5µm.

Also, the changes between stages are continuous. Thus, few cells incorrectly classified presented main features of a given phase combined with some features of the previous or following phase. Our algorithm, sometimes, fail to correctly detect these cells that show parameters close to the thresholds considered in the routine. When manually classifying these cells, we used the criteria described in the manuscript for splitting cells between contiguous phases (Materials and Methods: Cell cycle classification with PCNA-RFP).

The performance of this routine, and of other quantitative methods described in the literature (Schonenberger et al., 2015), are not better than the manual classification widely used by the scientific community employing PCNA distribution (Barr et al., 2016, Pomerening et al., 2008, Leonhardt et al., 2000, Sporbert et al., 2002, Velasquez et al., 2022, Xie and Bankaitis, 2022, Velasquez et al., 2019, Dutta et al., 2019, Leung et al., 2011, Wilson et al., 2016). Indeed, the gold standard used for evaluating the performance of automate methods is the manual classification (e.g. (Schonenberger et al., 2015)). To our knowledge, there are no other methods described in the literature that provide a clear

advantage (in terms of precision) over the manual classification methodology widely used by the scientific community.

Additional References

Barr, A. R., Heldt, F. S., Zhang, T., Bakal, C. & Novak, B. 2016. A Dynamical Framework for the All-or-None G1/S Transition. Cell Syst*,* 2**,** 27-37.

Dutta, P., Islam, S., Choppara, S., Sengupta, P., Kumar, A., Kumar, A., Wani, M. R., Chatterjee, S. & Santra, M. K. 2019. The tumor suppressor FBXO31 preserves genomic integrity by regulating DNA replication and segregation through precise control of cyclin A levels. J Biol Chem*,* 294**,** 14879-14895.

Leonhardt, H., Rahn, H. P., Weinzierl, P., Sporbert, A., Cremer, T., Zink, D. & Cardoso, M. C. 2000. Dynamics of DNA replication factories in living cells. J Cell Biol*,* 149**,** 271-80.

Leung, L., Klopper, A. V., Grill, S. W., Harris, W. A. & Norden, C. 2011. Apical migration of nuclei during G2 is a prerequisite for all nuclear motion in zebrafish neuroepithelia. Development*,* 138**,** 5003-13.

Pomerening, J. R., Ubersax, J. A. & Ferrell, J. E., Jr. 2008. Rapid cycling and precocious termination of G1 phase in cells expressing CDK1AF. Mol Biol Cell*,* 19**,** 3426-41.

Schonenberger, F., Deutzmann, A., Ferrando-May, E. & Merhof, D. 2015. Discrimination of cell cycle phases in PCNA-immunolabeled cells. BMC Bioinformatics*,* 16**,** 180.

Sporbert, A., Gahl, A., Ankerhold, R., Leonhardt, H. & Cardoso, M. C. 2002. DNA polymerase clamp shows little turnover at established replication sites but sequential de novo assembly at adjacent origin clusters. Molecular cell*,* 10**,** 1355-65.

Velasquez, Z. D., Conejeros, I., Larrazabal, C., Kerner, K., Hermosilla, C. & Taubert, A. 2019. Toxoplasma gondii-induced host cellular cell cycle dysregulation is linked to chromosome missegregation and cytokinesis failure in primary endothelial host cells. Sci Rep*,* 9**,** 12496.

Velasquez, Z. D., Rojas-Baron, L., Larrazabal, C., Salierno, M., Gartner, U., Pervizaj-Oruqaj, L., Herold, S., Hermosilla, C. & Taubert, A. 2022. Neospora caninum Infection Triggers S-phase Arrest and Alters Nuclear Characteristics in Primary Bovine Endothelial Host Cells. Front Cell Dev Biol*,* 10**,** 946335.

Wilson, K. A., Elefanty, A. G., Stanley, E. G. & Gilbert, D. M. 2016. Spatio-temporal re-organization of replication foci accompanies replication domain consolidation during human pluripotent stem cell lineage specification. Cell Cycle*,* 15**,** 2464-75.

Xie, Z. & Bankaitis, V. A. 2022. Phosphatidylinositol transfer protein/planar cell polarity axis regulates neocortical morphogenesis by supporting interkinetic nuclear migration. Cell Rep*,* 39**,** 110869.
