# Supplementary material for: The dynamical organization of the core pluripotency transcription factors responds to differentiation cues in early S-phase
Source: Front Cell Dev Biol. 2023 May 4;11:1125015. doi: 10.3389/fcell.2023.1125015 (PMC10192714; doi:10.3389/fcell.2023.1125015)
Supplement: Supplementary file 2 [file DataSheet1.pdf]

1. Supplementary Figures

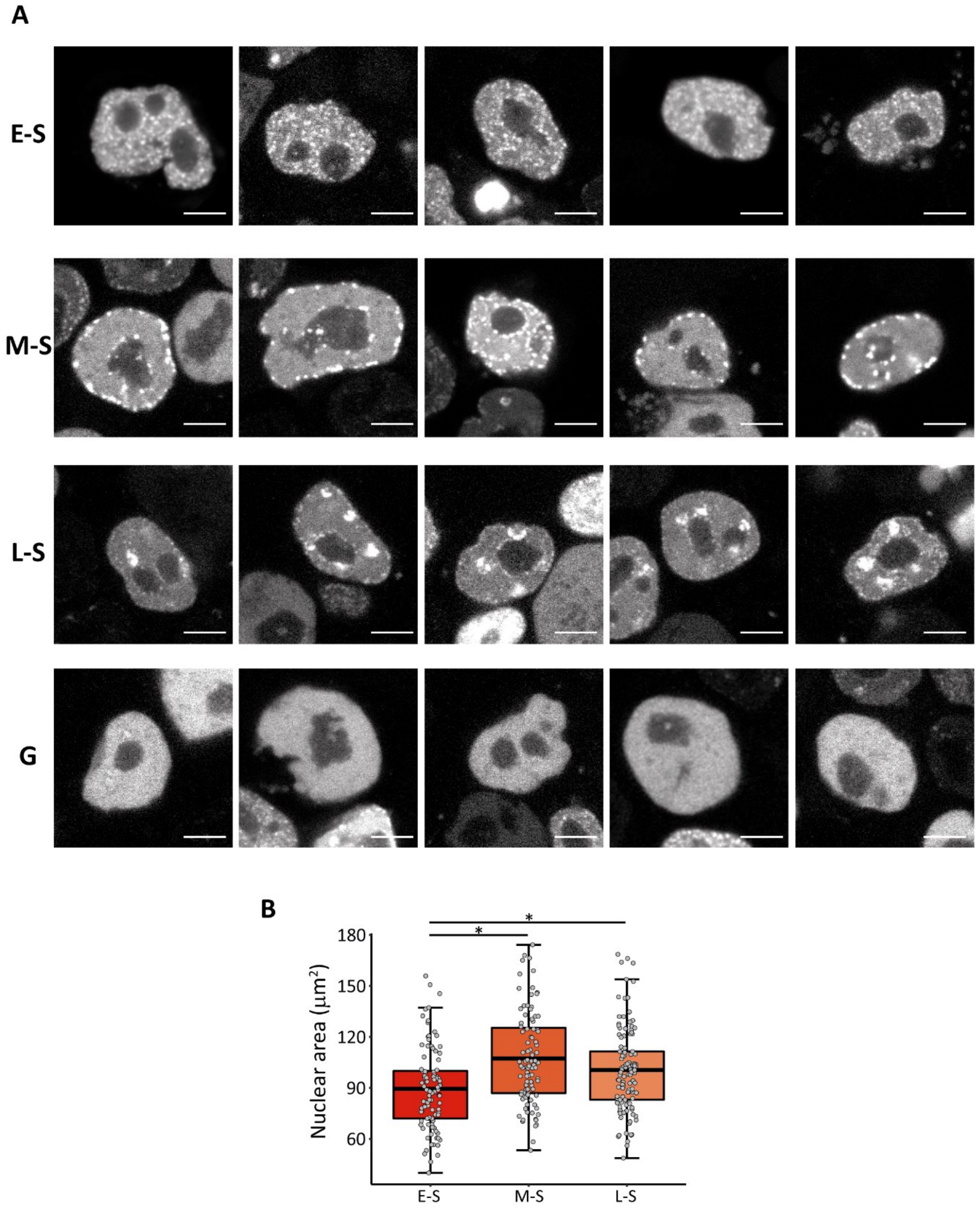

Supplementary Figure 1. (**A**) Representative confocal images of PCNA-RFP in E-S, M-S, L-S or G cells. Scale bars: 5 µm. (**B**) Nuclear area obtained from confocal images of E-S, M-S and L-S cells (dark to light red). The thick, black lines in boxplots represent the mean values. *P-value < 0.05.

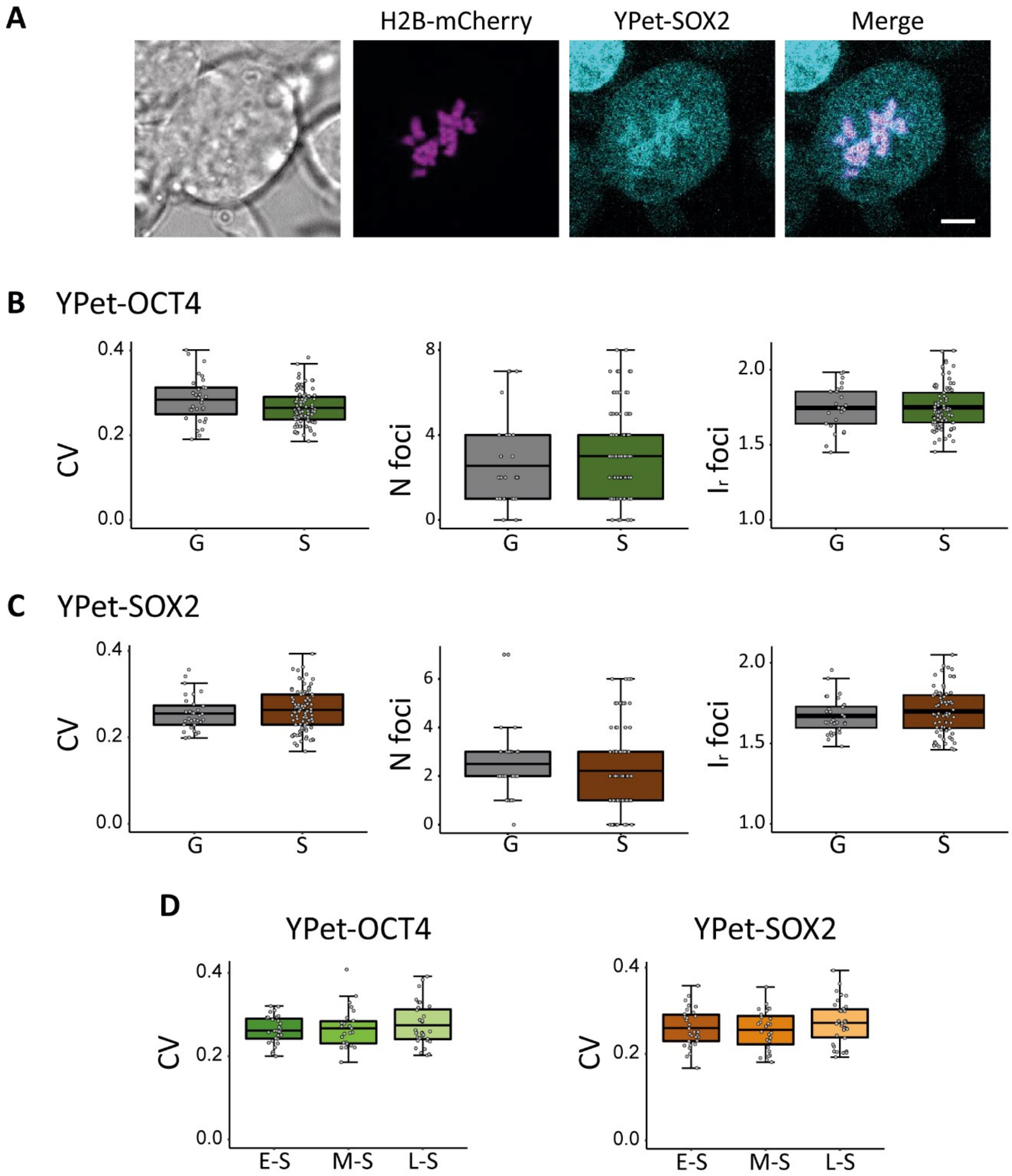

Supplementary Figure 2. (**A**) Representative confocal image of an ESC expressing YPet-SOX2 and H2B-mCherry in mitosis. Scale bar: 5 µm. (**B**) YPet-OCT4 and (**C**) YPet-SOX2 values of CV (left), N foci (middle) and I_r foci (right) determined in cells in G (gray) and S (color) phases. (**D**) YPet-OCT4 (left) and YPet-SOX2 (right) values of CV in E-S, M-S and L-S phases (dark to light color). The thick, black lines in the boxplots represent the mean values. *P-value<0.05. Number of analyzed cells: YPet-OCT4: 31 (G), 93 (S); YPet-SOX2: 32 (G), 92 (S).

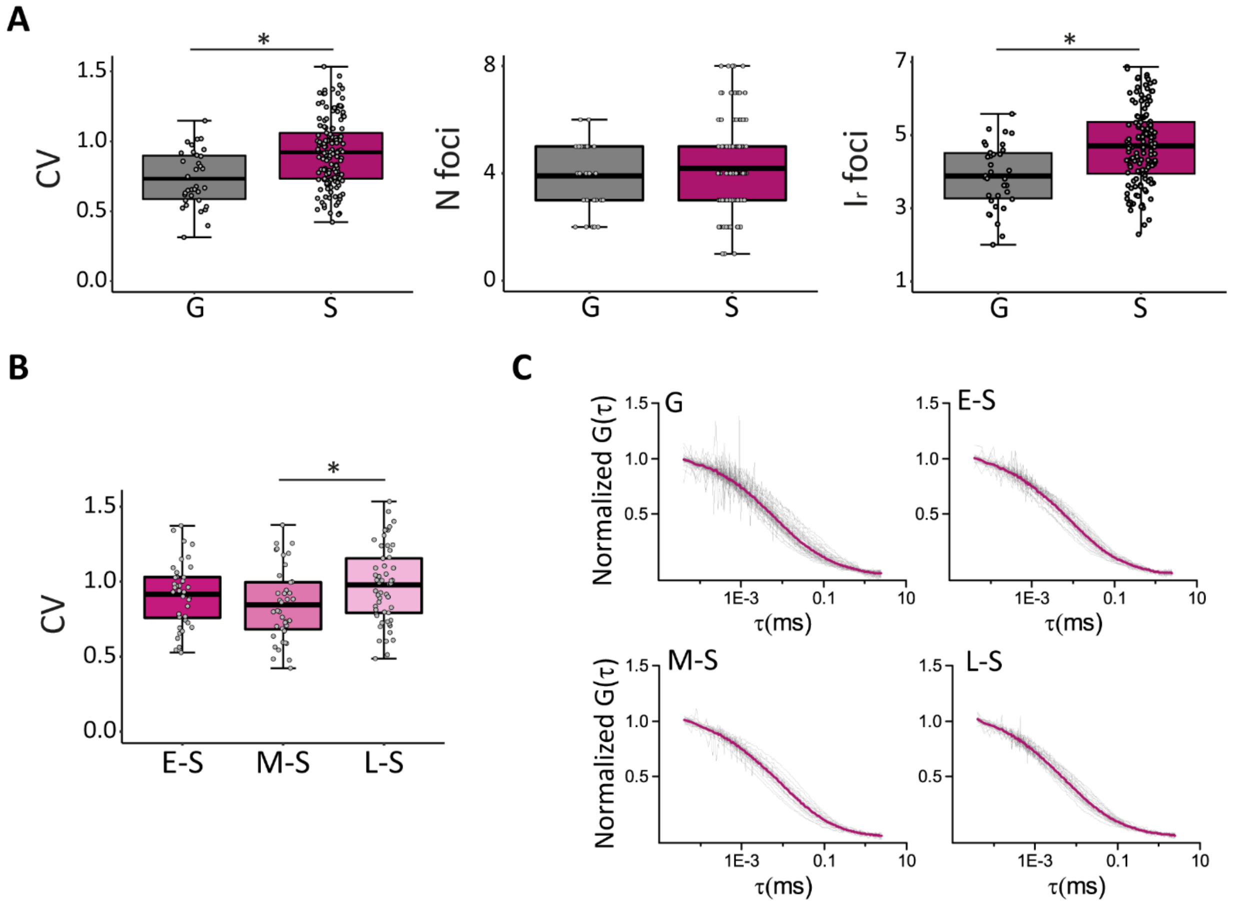

Supplementary Figure 3. (**A**) Values of CV (left), N foci (middle) and I_r foci (right) for ESCs transfected with HP1α-eGFP determined in cells in G (gray) and S (pink) phases. (**B**) CV values in E-S, M-S and L-S cells (dark to light color). (**C**) ACFs and mean ACF (pink) obtained for HP1α-eGFP during G, E-S, M-S or L-S phases. The thick, black lines in the boxplots represent the mean values. *P-value<0.05. Number of analyzed cells: 34 (G), 138 (S).

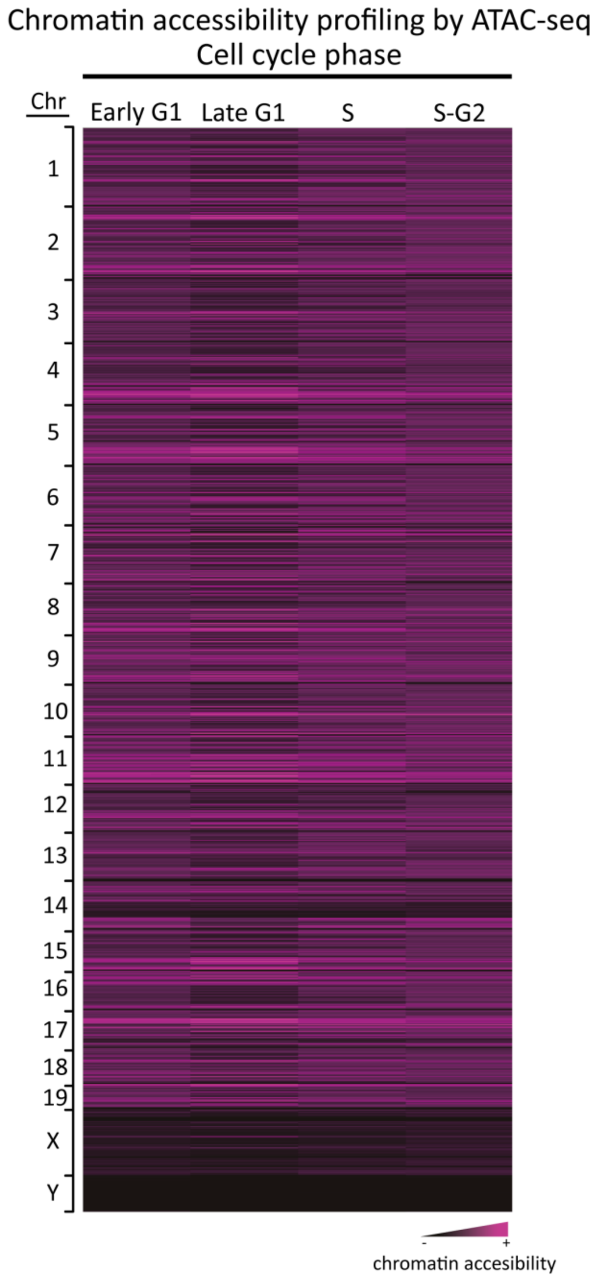

Supplementary Figure 4. Profile heatmap showing density of mapped ATAC-seq reads throughout the genome from publicly available experimental data of ESCs at Early G1, Late G1, S and S-G2 cell cycle phases [1]. Regions were ordered by chromosome (Chr) number, indicated in the left. Read counts were visualized using the Integrative Genomics Viewer (IGV) software [2]. The gradient black-to-violet color indicates low-to-high counts in the corresponding chromosomic region. Data normalization, transformation and annotation methods were performed by the authors of the original dataset and are available at Gene Expression Omnibus project profile (dataset accession number: GSE134673).

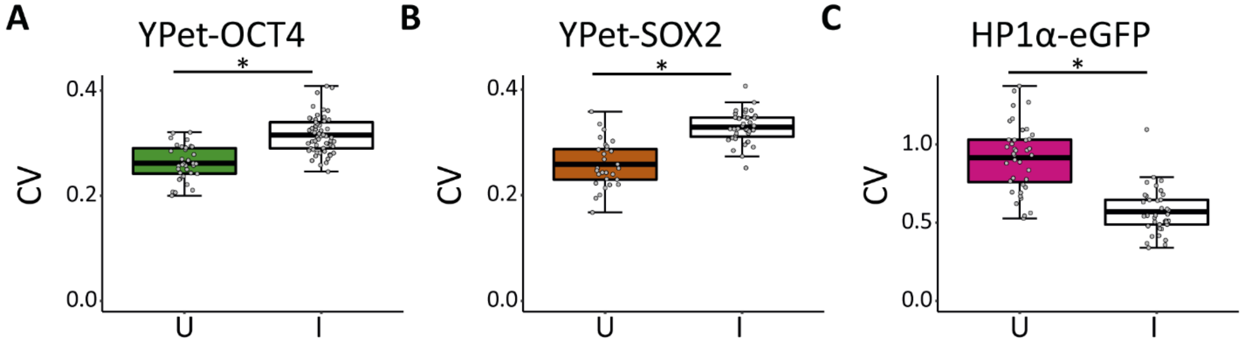

Supplementary Figure 5. (**A**) YPet-OCT4, (**B**) YPet-SOX2 and (**C**) HP1α-eGFP values of CV (filled and empty boxplots for U and I cells, respectively). The thick, black lines in the boxplots represent the mean values. *P-value<0.05.

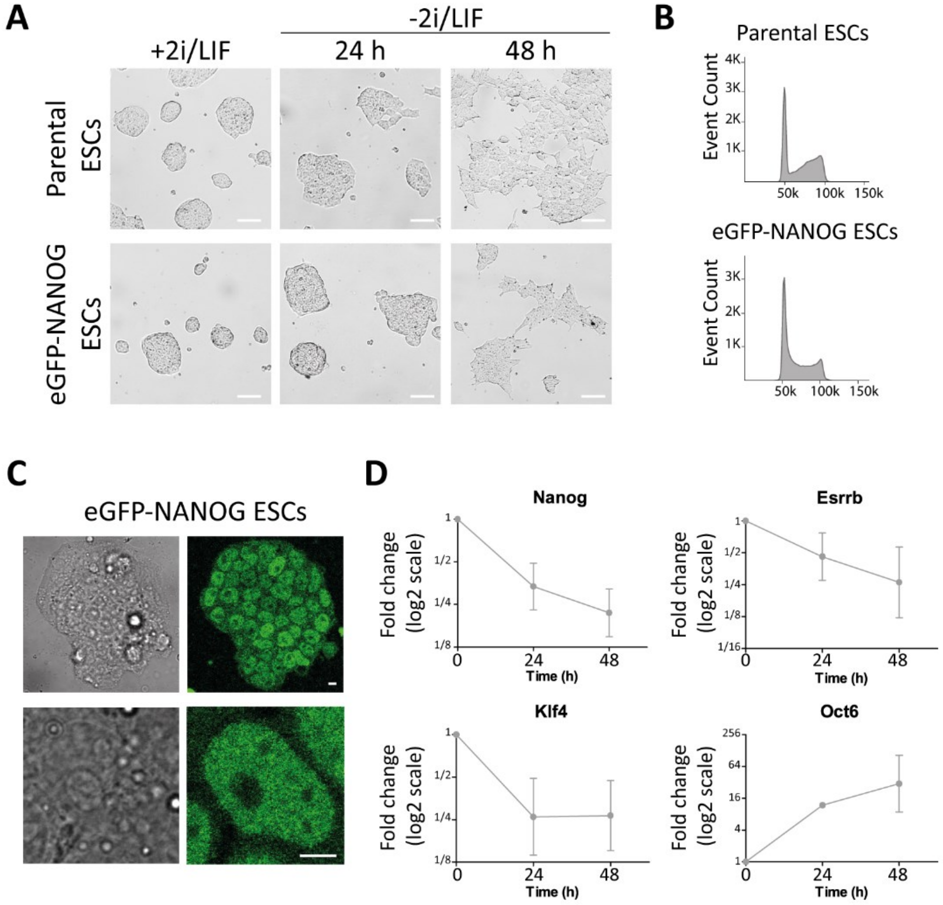

Supplementary Figure 6. (**A**) Representative images showing the characteristic morphology of the parental and eGFP-NANOG ESCs colonies cultured in undifferentiated conditions (+2i/LIF) and after 24 or 48 h of differentiation induction (-2i/LIF). Scale bars: 50 µm (**B**) Cell cycle

distribution of parental and eGFP-NANOG ESC lines after propidium iodide staining and flow cytometry analysis. (**C**) Representative confocal images showing eGFP-NANOG expression. Scale bars: 5µm. (**D**) RT-qPCR analysis of the indicated genes in eGFP-NANOG ESCs cultured in undifferentiated medium (0 h) or without 2i and LIF for 24 or 48 h. Results are presented as means ± SEM and plotted in log2 scale, relative to the undifferentiated condition.

2. Supplementary Table

Supplementary Table S1: RT-qPCR primer sequences

Name	Note	Sequence (5'-3')
**Gapdh**	Forward	TGCCAAGGCTGTGGGCAAGG
	Reverse	CGAAGGTGGAAGAGTGGG
**Esrrb**	Forward	GAACACTCTCGCCTGGTAGG
	Reverse	CGCCTCCAGGTTCTCAATGT
**Klf4**	Forward	TACCCTCCTTTCCTGCCAGA
	Reverse	TTTGCCACAGCCTGCATAGT
**Nanog**	Forward	AGGGTCTGCTACTGAGATGCTCTG
	Reverse	CAACCACTGGTTTTTCTGCCACCG
**Oct6**	Forward	CTCACCTTTTCTCCGGGCTT
	Reverse	ATACACAGATGCGGCTCTCG

Supplementary references

[1] E.T. Friman, C. Deluz, A.C. Meireles-Filho, S. Govindan, V. Gardeux, B. Deplancke, and D.M. Suter, Dynamic regulation of chromatin accessibility by pluripotency transcription factors across the cell cycle. Elife 8 (2019).

[2] J.T. Robinson, H. Thorvaldsdottir, W. Winckler, M. Guttman, E.S. Lander, G. Getz, and J.P. Mesirov, Integrative genomics viewer. Nat Biotechnol 29 (2011) 24-6.
